# The mechanism of *Bacillus subtilis* regulating the underground system and bamboo shoot quality of Lei bamboo (*Phyllostachys praecox*) under bamboo charcoal addition

**DOI:** 10.3389/fpls.2025.1703536

**Published:** 2025-11-27

**Authors:** KaiWen Huang, YuWen Peng, ZhenMing Wang, HongYang Luo, ZuoRong Wen, YuFang Bi, AnKe Wang, JiaXing Qin, XuHua Du

**Affiliations:** 1 China National Bamboo Research Center, Key Laboratory of State Forestry and Grassland Administration on Bamboo Forest Ecology and Resource Utilization, Hangzhou, China; 2 College of Forestry, Nanjing Forestry University, Nanjing, China; 3 Pujiang County Forest Resources Management Station, Jinhua, China; 4 Dongyang Forestry Seedling Management Station, Jinhua, China; 5 Nenya Family Farm, Yuhang Sub-district, Yuhang District, Hangzhou, China

**Keywords:** *Bacillus subtilis*, bamboo charcoal, Lei bamboo, endogenous hormone, bamboo shoots

## Abstract

**Introduction:**

Lei bamboo (Phyllostachys praecox) is widely cultivated due to its early-season bamboo shoots, but the mechanisms linking its underground system regulation and shoot quality remain unclear.

**Methods:**

This study assessed the impact of Bacillus subtilis (T1, 250 kg·hm-2), bamboo charcoal (T2, 1500 kg·hm-2), and their combination (T3) on soil chemistry, whips and roots morphology and biomass, hormone regulation, and the biochemical quality of bamboo shoots.

**Results:**

Treatment T1 notably increased soil total organic carbon and nitrogen, promoted whip development and root activity. These improvements were linked to elevated levels of GA, IAA, and CTK hormones and reduced ABA levels, which led to higher starch, soluble sugar, protein, and total amino acid contents in the bamboo shoots. Treatment T2 increased biomass of whips and roots, but also raised ABA and organic acids, improved protein and amino acids, while decreasing starch and sugar accumulation. The combined treatment T3 showed antagonistic effects, lowering amino acid content. The comprehensive evaluation ranking of bamboo shoot quality was T1 > T2 > T3 > CK.

**Discussion:**

These results reveal a new “soil chemistry-hormone-morphology and biomass-quality” cascade mechanism, and emphasize Bacillus subtilis and bamboo charcoal as promising fertilizers for producing sweet, crisp, and flavorful bamboo shoots. For practical application, it is advised that farmers apply 250 kg·hm-2 Bacillus subtilis as the fertilizer choice for the production of sweet, crisp and juicy bamboo shoots, and 1500 kg·hm-2 bamboo charcoal as the fertilizer choice for the production of bamboo shoots with unique flavors before mulching each November to enhance bamboo shoot quality and supply high-value raw materials for the bamboo industry.

## 1 Introduction

*Bacillus subtilis* is a beneficial soil bacterium that is extensively utilized to enhance crop quality and yield (Mehmood et al., 2023; Lv et al., 2025). Its positive effects are observed across a variety of crops and cultivation conditions, primarily through the improvement of soil health (Zhao, S et al., 2025), facilitation of plant growth, and augmentation of resistance to stress and disease (Gayathri et al., 2025), thereby enhancing overall crop quality. Empirical studies have demonstrated that *Bacillus subtilis* can significantly increase plant height, biomass, and yield in crops such as sweet corn (Qin et al., 2023), tomato (Sarti et al., 2024), soybean (Haleema et al., 2025), and cucumber Wang, W et al. (2023), contingent upon the specific crop and environmental conditions. This bacterium enhances the availability of essential soil nutrients, including nitrogen, phosphorus, and potassium, which results in improved nutrient uptake by plants and the production of higher quality agricultural products (Jiang et al., 2024; Zhou et al., 2022). Furthermore, *Bacillus subtilis* aids crops in coping with water, salt, and salinity stress by enhancing physiological characteristics, such as increased proline levels, elevated antioxidant enzyme activity, and reduced oxidative damage, as well as improving soil water retention (Lv et al., 2025). Additionally, it reduces the ecological harm caused by relying on traditional chemical fertilizers, and mitigates the impact of soil-borne pathogens and induces systemic resistance, thereby decreasing the incidence and severity of diseases, which ultimately contributes to the production of healthier and higher-quality crops (Blake et al., 2021; Amin and Ahmed, 2023; Ramakrishna et al., 2022).Recent studies on how soil conditioners regulate soil microbial communities to enhance soil fertility have offered more detailed mechanistic understanding. For instance, in the saline-alkali soils of the Yellow River Delta, applying external conditioners notably increased the soil organic carbon content and altered the bacterial community structure. This beneficial effect is closely linked to the improved carbon-nitrogen cycling driven by microorganisms (Zhao, H et al., 2025). These results align well with soil improvements facilitated by *Bacillus subtilis*, highlighting the significant potential of boosting soil fertility through managing the soil microenvironment.

Bamboo charcoal is a solid material produced through the pyrolysis of bamboo, conducted under conditions of elevated temperature and limited oxygen availability. Its application in agriculture has gained traction due to its capacity to enhance soil quality and promote crop health. Specifically, bamboo charcoal contributes to the enrichment of soil nutrients, restores soil vitality (Fallah et al., 2023), fosters beneficial microbial populations, mitigates the presence of detrimental substances, and enhances plant growth and resilience, ultimately resulting in improved crop quality and yield. It has been observed that bamboo charcoal elevates soil pH, increases organic matter content, and enhances the availability of critical nutrients such as nitrogen, phosphorus, and potassium, all of which are vital for optimal crop development (Wu et al., 2022). Furthermore, bamboo charcoal promotes the diversity and abundance of beneficial soil bacteria, which play a significant role in nutrient cycling and overall plant health (Chen et al., 2023). Additionally, it stimulates the synthesis of secondary metabolites in plants, including flavonoids, terpenoids, and phenolic acids, which bolster plant defenses against pests and diseases (Ren et al., 2023). Empirical studies have demonstrated that bamboo charcoal can enhance crop quality. For instance, in cassava leaves, it has been shown to increase levels of crude protein, fiber, and digestibility while simultaneously reducing harmful phytochemicals (Siska et al., 2024). In tobacco plants, bamboo charcoal aids in recovery from pesticide-induced stress and improves leaf quality (Yin et al., 2023).

Bamboo shoots have long been used as direct dietary supplements or processed to obtain bamboo drape, bamboo vinegar and bamboo shoot oil, which are rich in a variety of fatty acids, phytosterols and vitamins used in medical products, health supplements or fertilizers (Ankush et al., 2023). Lei bamboo (*Phyllostachys praecox*) is recognized as a highly suitable bamboo species for the cultivation of bamboo shoots, characterized by a relatively short cultivation period, early emergence of shoots, an extended harvesting season, and high yield potential (Qian et al., 2025). The bamboo shoots exhibit a high content of protein and amino acids while maintaining low levels of fat and crude fiber, which contributes to their popularity among consumers (Hu et al., 2023). The underground whip root system of Lei bamboo serves as a critical organ for the synthesis of organic compounds and physiologically active substances. This root system plays a significant role in influencing the growth and reproductive capacity of bamboo forests through mechanisms such as whip expansion and shoot development, thereby determining the regeneration and renewal potential of Lei bamboo populations (Umemura and Takenaka, 2014).

Plant hormones are essential regulators of various aspects of plant growth, development, and responses to environmental stressors. By modulating physiological processes and enhancing stress tolerance, these hormones are instrumental in improving plant quality, which encompasses nutritional value, yield, and resilience. Hormones such as auxins, gibberellins, cytokinins, and abscisic acid are involved in coordinating cell division, elongation, root and shoot growth, flowering, and fruit ripening, thereby directly impacting plant structure and productivity (Thapa et al., 2024; Guleria et al., 2021; Bigatton et al., 2024). Appropriate hormone treatments can enhance the concentration of bioactive compounds (including phenolics, pigments, and vitamins), increase antioxidant capacity, and promote health benefits in plants such as sprouts, ultimately improving the nutritional quality of crops (Guleria et al., 2021; Zou et al., 2024).

Historically, research has concentrated on the underground rhizome and the regulation of endogenous hormones in the physiological growth processes of Lei bamboo. This includes investigations into how underground rhizomes respond to soil aeration and acidity (Deng et al., 2023), as well as the effects of plant hormones on the growth and flowering of Lei bamboo (Lu et al., 2012). However, there remains a paucity of studies examining the mechanisms by which the interactions between underground system and hormonal changes influence the quality of bamboo shoots. Although the effects of *Bacillus subtilis* and bamboo charcoal on other crops have been well documented, their effects on bamboo shoots and the specific mechanism have not been well studied. Past research has focused on *Bacillus subtilis* regulating bamboo growth, primarily through the production of growth-promoting hormones (Belincanta et al., 2021), aiding in bioremediation (Saini and Arya, 2016), and enhancing soil health through enzyme production (Li et al., 2024). The objective of this research is to examine the impact of *Bacillus subtilis* and bamboo charcoal on the morphology, biomass and hormonal levels of the underground system of Lei bamboo, study the effects of these changes on the quality of its shoots. This research investigates the mechanisms by which the quality of bamboo shoots is enhanced and addresses the inadequacies in quality-focused regulatory technologies for bamboo shoots in the industry.

## 2 Materials and methods

### 2.1 Site description

**Figure 1:**
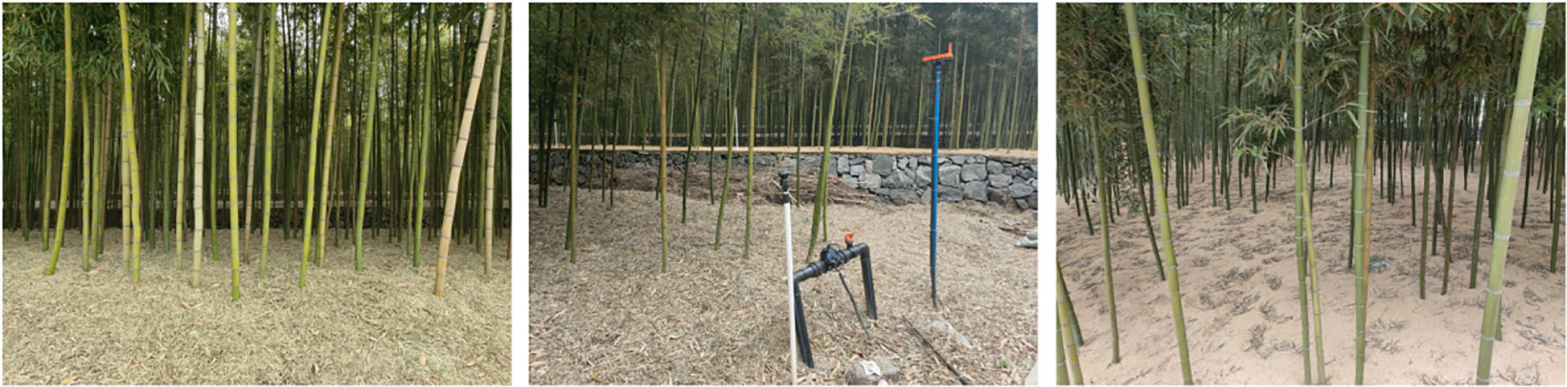
Overview of intensively Lei bamboo (*Phyllostachys praecox*) forest at the study site.

The experimental site is situated in Xianzhai Village, within the Yuhang District of Hangzhou City, Zhejiang Province (30°18'35"N, 119°53'3"E). This site features an intensively managed Lei bamboo (*Phyllostachys praecox*) forest that has been operational for approximately 15 years. The forest exhibits a continuous distribution of *Phyllostachys praecox*, with an average diameter at breast height of 3.50 ± 0.94 cm and a density of 14,903 ± 335 plants per hectare. The age structure of the forest is represented by a ratio of approximately 3:4:2:1 for the age classes of 1 year, 2 years, 3 years, and 4 years, respectively (**Figure 1**). The basic physical and chemical properties of soil were as follows: pH 4.130 ± 0.040, total organic carbon 58.668 ± 0.767g·kg^-1^, total nitrogen 4.510 ± 0.080g·kg^-1^, total phosphorus 1.429 ± 0.057g·kg^-1^, total potassium 9.793 ± 0.315g·kg^-1^, available nitrogen 0.532 ± 0.010g·kg^-1^, available phosphorus 222.989 ± 32.975mg·kg^-1^, and available potassium 303.730 ± 11.930mg·kg^-1^.

### 2.2 Test materials

The *Bacillus subtilis* B10 strain utilized in this experiment, characterized by an effective viable bacterial count of at least 285 million per gram and a contamination rate not exceeding 10%, was supplied by the laboratory of Li Weifen at Zhejiang University, China. Additionally, the bamboo charcoal, with a particle size of 40–80 mesh, was sourced from Suichang Shenlonggu Charcoal Industry Co., Ltd.

### 2.3 Experimental design

#### 2.3.1 Pre-experiment

Because bamboo charcoal has a large specific surface area and strong adsorption properties, it can either promote or inhibit *Bacillus subtilis* when added to soil. To assess how well these two interact in soil and to identify the best mixing ratio that maximizes the bacteria's viability in field use, we conducted this preliminary experiment. We tested five different ratios (1: 2, 1: 4, 1: 6, 1: 8, and 1: 10) to thoroughly explore the possible nonlinear impact of bamboo charcoal dosage on bacterial survival. The experiment included five treatment groups, each with six replicates, totaling 30 samples. 100g of sterile soil and 50ml of sterile water were prepared in a glass tissue culture flask for each sample to simulate 50% of the field water holding capacity. *Bacillus subtilis* and bamboo charcoal were the experimental variables. Details of the treatment types are provided in **Table 1**. After incubating the samples at 30°C for 2 and 6 days, 1 gram from each sample was taken and subjected to serial dilution followed by the plate coating method. The dilution that yielded the clearest colony count was used to observe the number of *Bacillus subtilis* B10 colonies in 5 × 10–^10^ grams of bacterial agent.

**Table 1:** Effect of bamboo charcoal application rate on the number of *Bacillus subtilis* colonies.

Treatment	Experimental setting	Proportion	Number of colonies (2d)	Number of colonies (6d)
A	0.5g of *Bacillus subtilis* + 1g of bamboo charcoal	1 : 2	84.20 ± 16.27b	226.60 ± 65.68b
B	0.5g of *Bacillus subtilis* + 2g of bamboo charcoal	1 :4	95.16 ± 18.24b	269.54 ± 60.32c
C	0.5g of *Bacillus subtilis* + 3g of bamboo charcoal	1 : 6	150.00 ± 26.04a	313.80 ± 37.56a
D	0.5g of *Bacillus subtilis* + 4g of bamboo charcoal	1 : 8	136.54 ± 32.46c	258.79 ± 46.74c
E	0.5g of *Bacillus subtilis* + 5g of bamboo charcoal	1 : 10	104.20 ± 28.31b	243.40 ± 57.28bc

The data in the table is “average ± standard deviation”. Different lowercase letters in the same column indicate significant (*p* < 0.05) differences among different fertilization treatments.

The preliminary experimental results (**Table 1**) clearly demonstrated that the proportion of bamboo charcoal had a significant impact on the number of *Bacillus subtilis* colonies. After 2 and 6 days of incubation, the 1: 6 treatment group (C) showed a significantly (*p* < 0.05) higher colony count compared to all other groups, indicating that this ratio offers the most favorable microenvironment for *Bacillus subtilis*. Notably, when the ratio was below 1:6 (such as 1:4 and 1:2), the colony numbers decreased as the amount of bamboo charcoal decreased, suggesting that insufficient bamboo charcoal fails to provide enough attachment sites and physical protection for the bacteria. Conversely, when the ratio exceeded 1:6 (for example, 1:8 and 1:10), colony numbers also declined, implying that excessive bamboo charcoal might physically trap bacterial cells or adsorb growth-promoting substances they secrete due to its strong adsorption capacity, thereby inhibiting growth. Thus, the data clearly identified 1:6 as the optimal ratio, providing a solid foundation for subsequent field trials. Using this optimal ratio, we expanded the application to a field scale. The dosage of *Bacillus subtilis* used in the field trials (250 kg·hm^-2^) was based on the standard recommended amount for use in the Lei bamboo forest. Correspondingly, the bamboo charcoal application rate was calculated as 250 kg × 6 = 1500 kg·hm^-2^. Thus, the fundamental reasoning behind the 1:6 field ratio applied in this study is not based on traditional nutrient equivalence but is intended to replicate the optimal microbial activity and survival conditions identified in preliminary experiments at the field level, ensuring that *Bacillus subtilis* can effectively survive and function in the rhizosphere.

#### 2.3.2 Field fertilization experiments

In November 2024, an experimental study was conducted in a Lei bamboo (*Phyllostachys praecox*) forest located in Yuhang, Hangzhou, Zhejiang Province, characterized by consistent site conditions, forest stand structure, and management practices. The base fertilizer applied was a compound fertilizer at a rate of 750 kg·hm^-2^, with a nutrient composition of N: P_2_O_5_: K_2_O = 15: 15: 15. *Bacillus subtilis* and bamboo charcoal were utilized as supplementary fertilizers. The experiment comprised four treatments, as detailed in **Table 2**. Each treatment was replicated five times, resulting in a total of 20 experimental plots. Each plot measured 10 m × 5 m, and a randomized block design was employed. To mitigate potential interference from adjacent bamboo rhizome stems, these plots were separated by stone walls and variations in elevation. Prior to the application of the covering materials, the forest land was thoroughly irrigated. The covering procedure involved layering: 4 cm of rice husk as the upper layer, 15 cm of bamboo leaves as the middle layer, and 6 cm of wheat bran as the lower layer, with the materials being added in three separate installments. These covering materials are commonly used traditional mulches in the cultivation of local Lei bamboo shoots. Their primary function is to create an insulating layer that increases and maintains soil temperature during winter, which helps promote early sprouting and higher yields of bamboo shoots. In this study, all treatments used the same materials, thicknesses, and application methods for mulching. Therefore, any differences observed between treatment groups can be confidently attributed to the effects of the different fertilization treatments (*Bacillus subtilis* and bamboo charcoal) rather than the covering materials themselves.

**Table 2:** Different fertilization treatments of Lei bamboo (*Phyllostachys praecox*) forest.

Treatment	Increase the types of fertilizers applied
CK	No addition
T1	*Bacillus subtilis* 250 kg·hm^-2^
T2	Bamboo charcoal 1500 kg·hm^-2^
T3	*Bacillus subtilis* 250 kg·hm^-2^ + bamboo charcoal 1500 kg·hm^-2^

### 2.4 Sample collection and index determination

#### 2.4.1 Sample collection

At the initiation of Lei bamboo (*Phyllostachys praecox*) shoot emergence period (March 2024), five vigorous and mature whips, each measuring 1 meter in length, were randomly selected from the central areas of various plots. The stems were gently shaken to dislodge larger soil particles. Subsequently, the roots of the stem segments were excised and rinsed with distilled water. Then they were dried using absorbent paper, wrapped in aluminum foil, labeled with numbers, and immediately frozen in liquid nitrogen before being stored at -80°C. The diameter of Lei bamboo whip was measured using a vernier caliper, and data were collected on the number of whip segments and buds on the 1-meter bamboo whip. The fresh weights of the bamboo whips and roots were recorded, after which the samples were blanched in an oven at 105°C for 30 minutes. They were subsequently dried at 70°C until a constant weight was achieved, at which point the dry weights were measured and the biomass was calculated.

During the peak production phase of Lei bamboo shoots (April 2024), five intact shoots exhibiting minimal individual variation and free from diseases, pests, and mechanical damage were randomly selected from diverse locations. Initially, the individual fresh weight, base diameter, and height of the bamboo shoots were promptly measured *in situ*. Subsequently, the unavailable stumps and sheaths of the bamboo shoots were excised, and their mass was recorded. This facilitated the calculation of the availability ratio and the length-to-thickness ratio of the shoots. The samples were then immediately placed in a dry ice incubator for transport back to the laboratory. In the laboratory, each bamboo shoot was bisected longitudinally from the tip to the base. One part was homogenized using a tissue homogenizer for the analysis of starch, soluble sugars, total acids, proteins, and amino acids. Another part was weighed and subsequently dried in an oven at 60 °C. The dry matter weight and water content ratio were then assessed. Following this, the dried material was ground and passed through a 100-mesh sieve to determine the dietary fiber content.

#### 2.4.2 Index determination

Soil pH was determined by pH meter (soil: water =1: 2.5); total carbon content was determined by elemental analyzer; total nitrogen content was determined by Kjeldahl method; total phosphorus content was determined by molybdenum antimony colorimetry. The analytical techniques employed for the quantification of starch and total soluble sugars utilize the anthrone colorimetric method. Protein content is assessed through the Kjeldahl nitrogen determination method, while total acid levels are measured using high-performance liquid chromatography. The quantification of amino acids is conducted *via* the ninhydrin colorimetric method. Dietary fiber is analyzed through both the Corrasone method and hydrochloric acid hydrolysis method. Root viability is evaluated using the TTC reduction method. Additionally, the contents of indoleacetic acid (IAA), gibberellin (GA), cytokinin (CTK), and abscisic acid (ABA) are determined through enzyme-linked immunosorbent assay (ELISA).

Root viability was determined by triphenyltetrazole chloride (TTC) reduction method. The procedure is as follows: Weigh 0.5 g of fresh root sample, submerge in 10 mL of equal volume mixture of 0.4% TTC solution and phosphate buffer (0.06 M, pH 7.0), and incubate for 3 h under dark conditions at 37°C. Subsequently, add 2 mL of 1 M sulfuric acid to terminate the reaction. The roots are removed, absorbed and ground to produce red triphenylmethane (TTF) by repeated dipping with ethyl acetate. The extract was combined and the volume was determined, and the absorbance was determined at 485 nm. The system without roots and the rest of the steps was used as a blank control. Root viability is expressed as the amount of TTF generated per unit of fresh heavy roots per unit time (μg TTФ·g^-^¹·h^-^¹).

The contents of starch and soluble total sugar were determined by anthraquinone colorimetry. For soluble sugars: 0.2 g of bamboo shoot homogenate was weighed, extracted in a 10 ml 80% ethanol water bath, the extract solution was mixed with anthradone-sulfuric acid reagent (0.2 g anthracene was dissolved in 100 mL of concentrated sulfuric acid prepared in an ice water bath), heated in a boiling water bath for 10 minutes, and colorimetriced at 620 nm after cooling. A standard curve was made with a glucose standard solution. For starch: the residue after ethanol extraction was hydrolyzed in a boiling water bath with 5 mL of perchloric acid (9.2 mol/L) for 15 minutes, and the glucose content was determined by the same anthracene colorimetric method, and the starch content was calculated by multiplying by the conversion factor of 0.9.

### 2.5 Data analysis

Data integration and processing were carried out using Excel, and statistical analyses were performed with SPSS Statistics v.22. One-way analysis of variance (ANOVA) was used to test treatment effects, followed by Tukey’s HSD *post hoc* test for multiple comparisons. To reduce the likelihood of false positives, p-values were adjusted using the false discovery rate (FDR) method. All data are presented as means ± standard deviation (SD). Statistical significance was set at *p* < 0.05. Graphs were generated using Origin 2022 and Chiplot.

## 3 Results

### 3.1 The influence of *Bacillus subtilis* and bamboo charcoal on soil chemical properties of Lei bamboo (*Phyllostachys praecox*) forest

Based on the results shown in **Table 3**, the soil pH in the Lei bamboo (*Phyllostachys praecox*) forest significantly (*p* < 0.005) increased following the T1, T2, and T3 treatments, with the greatest rise of 3.569% observed in the T2 treatment. Compared to CK, all three treatments significantly (*p* < 0.005) enhanced the total organic carbon (TOC) in the soil by 54.225%, 16.157%, and 25.376%, respectively. The total nitrogen (TN) content in the soil also showed significant (*p* < 0.005) increases of 43.666%, 9.165%, and 18.004% with T1, T2, and T3 treatments, respectively, compared to CK. Regarding total phosphorus (TP), soil levels rose after T1 and T3 treatments but declined following T2 treatment; however, these changes were not statistically significant.

**Table 3:** The chemical properties of the soil in Lei bamboo (*Phyllostachys praecox*) forest in different treatments.

Chemical properties	Treatments			
	CK	T1	T2	T3
pH	3.618 ± 0.008d	3.696 ± 0.013b	3.748 ± 0.011a	3.642 ± 0.008c
TOC (g·kg^-1^)	47.718 ± 1.932d	73.593 ± 3.445a	55.428 ± 2.088c	59.827 ± 2.115b
TN (g·kg^-1^)	5.510 ± 0.107d	7.916 ± 0.131a	6.015 ± 0.106c	6.502 ± 0.182b
TP (g·kg^-1^)	1.928 ± 0.119ab	2.101 ± 0.152a	1.826 ± 0.091b	2.112 ± 0.070a

The data in the table is “average ± standard deviation”. Different lowercase letters in the same column indicate significant (p < 0.05) differences among different fertilization treatments.

### 3.2 The influence of *Bacillus subtilis* and bamboo charcoal on the alterations in the underground system of Lei Bamboo (*Phyllostachys praecox*)

#### 3.2.1 Impacts of different treatments on the morphological and biomass characteristics in the underground system of Lei bamboo (*Phyllostachys praecox*)

The findings presented in **Table 4** indicate that, a statistical analysis of the number of whip segments and buds on a 1-meter bamboo whip revealed that both T1 and T3 treatments significantly (*p* < 0.005) enhanced these metrics. Specifically, the T1 treatment led to an increase of 9.753% in the number of whip segments and 2.197% in the number of buds, while the T3 treatment resulted in increases of 2.747% and 1.658%, respectively. Furthermore, all three treatments significantly (*p* < 0.005) improved both bamboo whip biomass and bamboo root biomass, with the following order of magnitude observed: T2 > T1 > T3 > CK. Additionally, the T1 and T3 treatments significantly (*p* < 0.005) enhanced the root vitality of Lei bamboo, with increases of 8.631% and 7.738%, respectively. In summary, the application of *Bacillus subtilis*, bamboo charcoal, and their combined application effectively increases both bamboo whip biomass and bamboo root biomass in Lei bamboo. Notably, the application of *Bacillus subtilis* individually, as well as the combined application, significantly enhances the number of whip segments, the number of buds, and the root vitality of Lei bamboo.

**Table 4:** The morphological and biomass characteristics of the underground system of Lei bamboo (*Phyllostachys praecox*) in different treatments.

Treatment	Diameter of bamboo Whip (cm)	The number of whip segments on a 1-meter bamboo whip	The number of whip buds on a 1-meter bamboo whip	Bamboo whip biomass (g·kg^-1^)	Bamboo root biomass (g·kg^-1^)	Root vitality (μg·min^-1^·g^-1^)
CK	1.846 ± 0.113a	5.424 ± 0.015c	5.007 ± 0.002c	440.078 ± 11.448c	197.475 ± 12.043b	0.336 ± 0.005b
T1	1.967 ± 0.160a	5.953 ± 0.008a	5.117 ± 0.008a	464.001 ± 13.027b	284.236 ± 65.010a	0.365 ± 0.007a
T2	2.031 ± 0.203a	5.413 ± 0.007c	5.007 ± 0.001c	500.463 ± 8.844a	286.747 ± 12.249a	0.343 ± 0.123b
T3	1.942 ± 0.266a	5.573 ± 0.006b	5.090 ± 0.002b	461.823 ± 7.869b	274.457 ± 7.590a	0.362 ± 0.146a

The data in the table is “average ± standard deviation”. Different lowercase letters in the same column indicate significant (*p* < 0.05) differences among different fertilization treatments.

#### 3.2.2 Impacts of different treatments on endogenous hormones in the underground system of Lei bamboo (*Phyllostachys praecox*)

**Figure 2:**
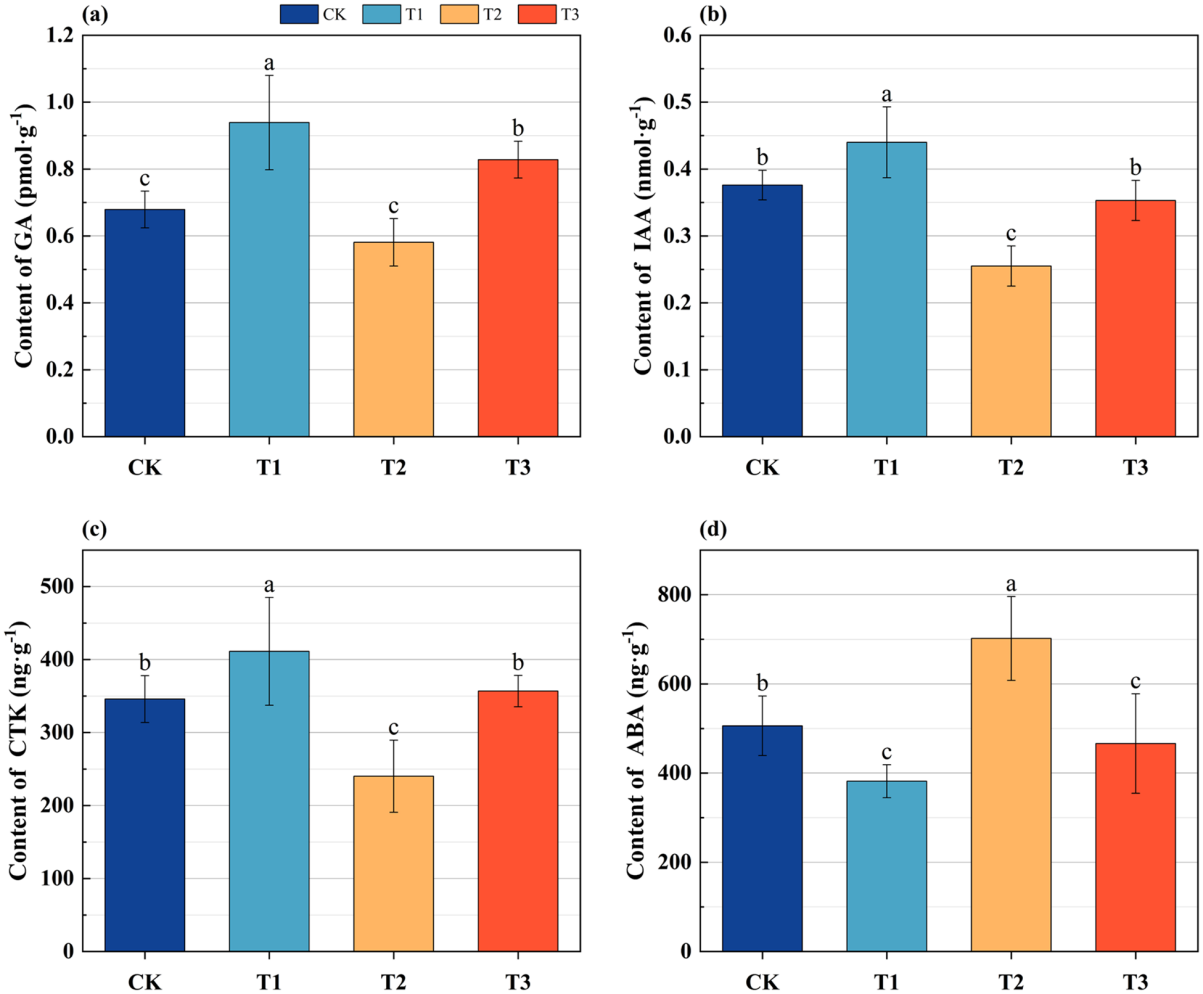
The endogenous hormone content of the underground system of Lei bamboo (*Phyllostachys praecox*) in different treatments. **(a)** gibberellins (GA); **(b)** indoleacetic acid (IAA); **(c)** cytokinins (CTK); **(d)** abscisic acid (ABA). Different letters indicate significant (*p* < 0.05) differences among different fertilization treatments.

In comparison to CK, the contents of gibberellin (GA) in the roots of Lei bamboo exhibited a significant (*p* < 0.005) increase following treatment with T1 and T3, with increases of 38.292% and 21.944%, respectively (**Figure 2a**). Additionally, the contents of indoleacetic acid (IAA) and cytokinin (CTK) showed significant (*p* < 0.005) increases of 17.021% and 18.873%, respectively, after T1 treatment. Conversely, following T2 treatment, these levels were significantly (*p* < 0.005) reduced by 32.181% and 30.570%, respectively (**Figures 2b**, **c**). The content of abscisic acid (ABA) significantly (*p* < 0.005) decreased by 24.561% and 7.845% after T1 and T3 treatments, respectively, while it significantly (*p* < 0.005) increased by 38.690% after T2 treatment (**Figure 2d**). These findings indicate that the application of *Bacillus subtilis* individually enhances the levels of GA, IAA, and CTK in the underground system of Lei bamboo while concurrently reducing ABA levels. In contrast, the application of bamboo charcoal individually leads to a decrease in GA, IAA, and CTK levels, accompanied by an increase in ABA content. The combined application results in an increase in GA content and a reduction in ABA levels.

#### 3.2.3 Correlation analysis of the morphology, biomass and endogenous hormones in the underground system of Lei bamboo (*Phyllostachys praecox*)

**Figure 3:**
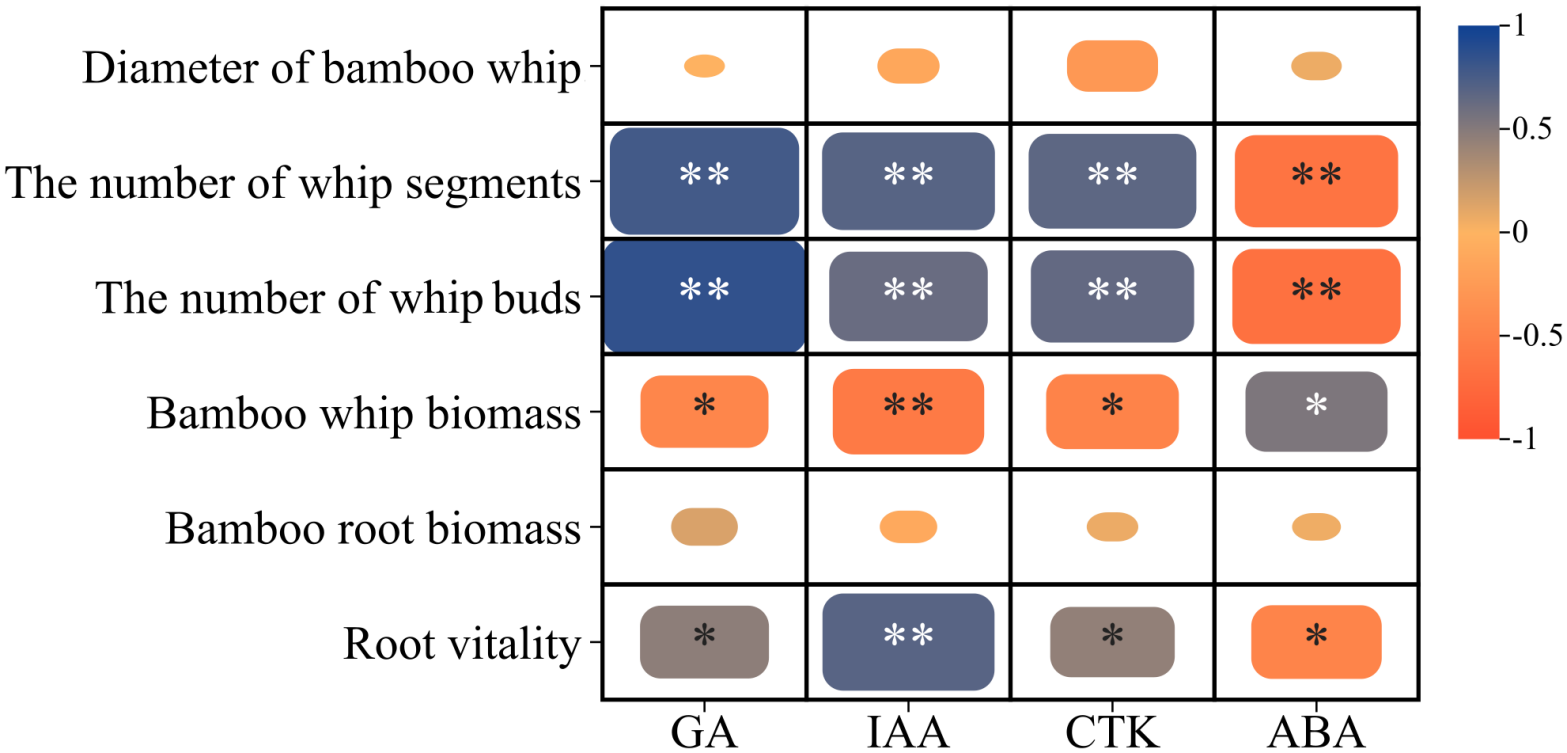
Correlation analysis of the morphology, biomass and endogenous hormones of the underground system of Lei bamboo (*Phyllostachys praecox*). Asterisks denote statistically significant differences between the groups, as measured by Mann–Whitney rank-sum test (**p < 0.01 and *p < 0.05).

The Pearson correlation analysis presented in **Figure 3** indicates that the number of whip segments on the 1-meter bamboo whip of Lei bamboo exhibited a highly significant positive correlation with GA (0.782), IAA (0.698), and CTK (0.672), while demonstrating a highly significant negative correlation with ABA (-0.649). Similarly, the number of buds on the 1-meter bamboo whip showed a highly significant positive correlation with GA (0.858), IAA (0.625), and CTK (0.649), alongside a highly significant negative correlation with ABA (-0.675). In contrast, the bamboo whip biomass was significantly positively correlated with ABA (0.540), while it was significantly negatively correlated with GA (-0.468) and CTK (-0.492), and exhibited a highly significant negative correlation with IAA (-0.589). Furthermore, root activity was significantly positively correlated with GA (0.473) and CTK (0.450) and showed a highly significant positive correlation with IAA (0.694). In summary, the number of whip segments and buds on the 1-meter bamboo whip, as well as the root activity of Lei bamboo, are positively correlated with GA, IAA, and CTK, while exhibiting negative correlations with ABA. Conversely, the bamboo whip biomass displays an opposing correlation pattern.

### 3.3 The influence of *Bacillus subtilis* and bamboo charcoal on the quality of Lei bamboo (*Phyllostachys praecox*) shoots

#### 3.3.1 Impacts of different treatments on the appearance quality of Lei bamboo (*Phyllostachys praecox*) shoots

The findings presented in **Table 5** indicate that the application of treatments T1, T2, and T3 led to an increase in the dry matter weight of the bamboo shoots relative to CK, with treatment T3 demonstrating a significant (*p* < 0.005) increase of 8.530%. Conversely, the water content ratio exhibited a decrease when compared to CK, with treatment T3 showing a significant (*p* < 0.005). reduction of 4.897% Furthermore, all three treatments significantly (*p* < 0.005) enhanced the length-to-thickness ratio of the bamboo shoots compared to CK, with increases of 22.702%, 5.244%, and 16.549% for T1, T2, and T3, respectively, establishing a hierarchy of T1 > T3 > T2 > CK. These results suggest that the application of *Bacillus subtilis*, bamboo charcoal, and their combination can positively influence the morphological characteristics and weight of Lei bamboo shoots, leading to increased availability ratios, dry matter weight, and length-to-thickness ratios, while simultaneously reducing water content.

**Table 5:** Appearance quality of Lei bamboo (*Phyllostachys praecox*) shoots in different treatments.

Treatment	Availability ratio (%)	Dry matter weight (g·kg^-1^)	Water content ratio (%)	Length-to-thickness ratio (%)
CK	53.714 ± 0.085a	111.471 ± 5.682b	88.853 ± 0.933a	30.376 ± 0.548d
T1	55.054 ± 0.068a	119.848 ± 1.921b	88.015 ± 2.06a	37.272 ± 0.652a
T2	56.006 ± 0.065a	112.592 ± 14.555b	88.741 ± 1.055a	31.969 ± 0.617c
T3	54.134 ± 0.088a	120.979 ± 18.378a	87.902 ± 1.045b	35.403 ± 0.604b

The data in the table is “average ± standard deviation”. Different lowercase letters in the same column indicate significant (*p* < 0.05) differences among different fertilization treatments.

#### 3.3.2 Impacts of different treatments on the nutritional quality of Lei bamboo (*Phyllostachys praecox*) shoots

An analysis of **Table 6** reveals that the starch content in the bamboo shoots of Lei bamboo subjected to treatments T1 and T3 exhibited a significant (*p* < 0.005) increase, with increases of 36.194% and 13.499%, respectively. Similarly, the soluble sugar content in bamboo shoots treated with T1 and T3 also showed a significant (*p* < 0.005) enhancement, with increases of 8.670% and 9.978%, respectively. Conversely, the starch and soluble sugar contents in bamboo shoots following T2 treatment were significantly (*p* < 0.005) reduced, with decreases of 53.663% and 5.661%, respectively. Furthermore, the total acid content in bamboo shoots treated with T1 and T3 was significantly (*p* < 0.005) lower than that of CK, with reductions of 30.357% and 27.574%, respectively. Conversely, following treatment T2, the total acid content exhibited a significant (*p* < 0.005) increase of 43.228%. The sugar-acid ratio exhibited a significant (*p* < 0.005) increase, with increases of 58.075% and 52.565% for T1 and T3, respectively. All treatments (T1, T2, and T3) significantly (*p* < 0.005) enhanced the protein content of bamboo shoots, with increases of 41.251%, 46.135%, and 31.448%, respectively, indicating a trend of T2 > T1 > T3 > CK. No significant differences were observed in dietary fiber content across the treatments. The analysis presented indicates that the application of *Bacillus subtilis* individually and their combined application can enhance the starch content, soluble sugar content, and sugar-acid ratio in Lei bamboo shoots. Furthermore, the application of bamboo charcoal individually has been shown to elevate the total acid content in bamboo shoots.

**Table 6:** Nutritional quality of Lei bamboo (*Phyllostachys praecox*) shoots in different treatments.

Treatment	Starch (mg·g^-1^)	Soluble sugar (mg·g^-1^)	Total acid (μg·g^-1^)	Sugar-acid ratio (%)	Protein (mg·g^-1^)	Dietary fiber (%)
CK	21.089 ± 0.415c	16.818 ± 0.372b	255.904 ± 27.111b	66.288 ± 6.932b	11.384 ± 0.950c	25.092 ± 4.447a
T1	28.722 ± 0.482a	18.276 ± 0.333a	178.218 ± 28.842c	104.785 ± 17.343a	16.080 ± 0.162a	24.491 ± 1.123a
T2	9.772 ± 0.174d	15.866 ± 0.473c	366.525 ± 28.075a	43.966 ± 4.917b	16.636 ± 0.610a	27.857 ± 2.320a
T3	23.936 ± 0.422b	18.496 ± 0.263a	185.340 ± 25.057c	101.132 ± 12.460a	14.964 ± 1.512b	26.018 ± 4.714a

The data in the table is “average ± standard deviation”. Different lowercase letters in the same column indicate significant (*p* < 0.05) differences among different fertilization treatments.

#### 3.3.3 Impacts of different treatments on amino acid content and proportion of Lei bamboo (*Phyllostachys praecox*) shoots

A total of 17 amino acids were identified in Lei bamboo shoots, which included 7 essential amino acids necessary for human health (valine, methionine, isoleucine, leucine, threonine, phenylalanine, lysine) and 2 semi-essential amino acids (cystine, tyrosine). As indicated in **Table 7**, the application of T1 and T2 treatments resulted in a significant (*p* < 0.005) increase in the content of total amino acids and essential amino acids in the bamboo shoots when compared to CK. Specifically, the total amino acids content increased by 26.266% and 8.987%, while the essential amino acids content rose by 36.598% and 10.046%, respectively. Conversely, following T3 treatment, there was a significant (*p* < 0.005) decrease in the total amino acids content and the essential amino acids content, with reductions of 8.841% and 5.544%, respectively. In conclusion, the application of *Bacillus subtilis* and bamboo charcoal individually enhances the total amino acids and essential amino acids content in the bamboo shoots of Lei bamboo. Notably, the combined application of both treatments produced an adverse effect.

**Table 7:** The amino acids components and contents of Lei bamboo (*Phyllostachys praecox*) shoots in different treatments.

Amino acids	Treatment			
	CK	T1	T2	T3
Aspartic acid (mg·g^-1^)	79.902 ± 17.269a	98.674 ± 21.353a	88.174 ± 17.446a	73.697 ± 21.448a
Glutamic acid (mg·g^-1^)	34.405 ± 9.254a	38.708 ± 6.187a	36.443 ± 8.879a	27.044 ± 7.176a
Serine (mg·g^-1^)	2.224 ± 0.541a	2.468 ± 0.383a	2.563 ± 0.511a	2.142 ± 0.505a
Arginine (mg·g^-1^)	2.715 ± 0.672b	7.840 ± 4.271a	3.011 ± 0.769b	5.359 ± 1.840ab
Glycine (mg·g^-1^)	2.560 ± 0.839b	4.382 ± 1.216a	2.795 ± 0.464b	2.818 ± 0.503b
Threonine (mg·g^-1^)	3.360 ± 0.477a	3.446 ± 0.380a	3.130 ± 0.258a	3.275 ± 0.495a
Proline (mg·g^-1^)	3.255 ± 0.592a	3.286 ± 0.588a	2.857 ± 0.568a	2.982 ± 0.695a
Alanine (mg·g^-1^)	9.109 ± 2.455a	9.860 ± 1.840a	9.953 ± 2.707a	8.800 ± 2.637a
Valine (mg·g^-1^)	3.264 ± 0.892ab	3.760 ± 0.877ab	4.091 ± 0.925a	2.651 ± 0.759b
Methionine (mg·g^-1^)	0.368 ± 0.054a	0.350 ± 0.113a	0.392 ± 0.057a	0.363 ± 0.101a
Cystine (mg·g^-1^)	0.409 ± 0.095a	0.484 ± 0.071a	0.475 ± 0.110a	0.429 ± 0.076a
Isoleucine (mg·g^-1^)	9.023 ± 6.329bc	16.660 ± 2.217a	5.681 ± 4.595c	11.800 ± 3.179ab
Leucine (mg·g^-1^)	5.371 ± 1.468a	7.545 ± 1.515a	5.702 ± 1.886a	5.387 ± 1.815a
Histidine (mg·g^-1^)	1.178 ± 0.378a	1.324 ± 0.250a	1.192 ± 0.223a	1.315 ± 0.320a
Phenylalanine (mg·g^-1^)	1.016 ± 0.413a	1.139 ± 0.441a	1.189 ± 0.364a	0.873 ± 0.243a
Lysine (mg·g^-1^)	29.797 ± 15.144a	38.403 ± 6.295a	37.258 ± 6.486a	24.956 ± 8.394a
Tyrosine (mg·g^-1^)	11.523 ± 4.063a	13.543 ± 6.708a	12.439 ± 3.725a	7.952 ± 2.753a
Total Amino Acids (mg·g^-1^)	199.478 ± 34.371b	251.873 ± 28.182a	217.345 ± 21.989a	181.842 ± 44.063c
Essential Amino Acids (mg·g^-1^)	52.199 ± 17.232b	71.303 ± 8.153a	57.443 ± 7.799a	49.305 ± 14.157c
Proportion of Essential Amino Acid (%)	25.729 ± 5.243a	28.463 ± 3.384a	26.402 ± 2.083a	26.792 ± 2.330a

The data in the table is “average ± standard deviation”. Different lowercase letters in the same row indicate significant (*p* < 0.05) differences among different fertilization treatments.

It is important to note, as shown in **Table 7**, that some amino acid measurements (such as aspartic acid, isoleucine, and lysine) exhibit large standard deviations. We believe this primarily reflects the natural individual variability present in bamboo shoots as biological samples. Although we carefully selected bamboo shoots with uniform appearance, it is normal for different specimens to have inherent fluctuations in their exact metabolite composition. This variation may result from subtle differences in the microenvironment, or the specific developmental stage of the shoots. However, by using appropriate experimental replicates (n=5) and thorough statistical analysis, we were able to identify a significant trend in the total and essential amino acid content between treatment groups, demonstrating that the fertilization treatment effect exceeded background variability and that our conclusions are reliable.

**Figure 4:**
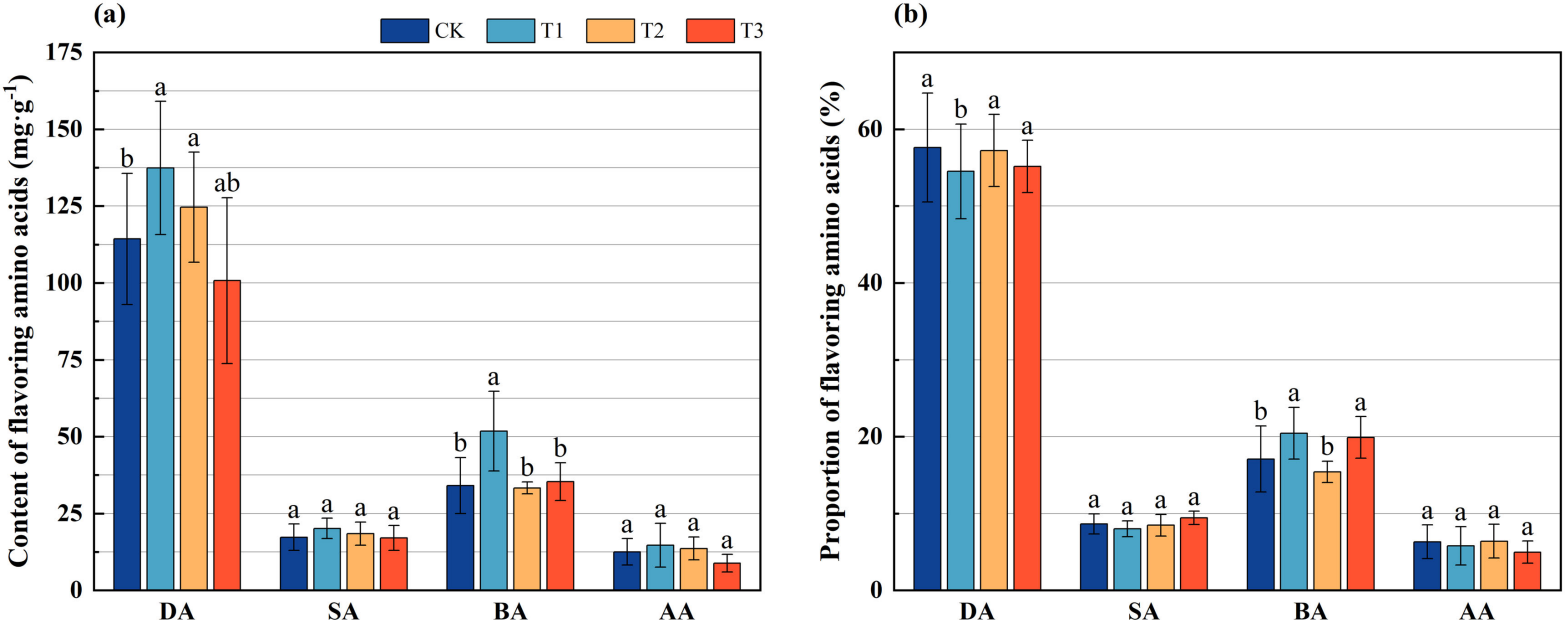
The content and proportion of flavoring amino acids in Lei bamboo (*Phyllostachys praecox*) shoots in different treatments. **(a)** Content of flavoring amino acids; **(b)** Proportion of flavoring amino acids. DA, Delicious amino acids, SA, Sweet amino acids; BA, Bitter amino acids; AA, Aromatic amino acids. Different letters indicate significant (*p* < 0.05) differences among different fertilization treatments.

Amino acids can be categorized based on their flavor profiles into four distinct groups: delicious amino acids (aspartic acid and glutamic acid), sweet amino acids (glycine, threonine, alanine, proline, and serine), bitter amino acids (valine, isoleucine, leucine, tyrosine, phenylalanine, histidine, and arginine), and aromatic amino acids (phenylalanine and tyrosine). An investigation into the flavoring amino acids present in the bamboo shoots of Lei bamboo indicated that the contents of delicious, sweet, bitter, and aromatic amino acids in the bamboo shoots subjected to treatment T1 were significantly higher than those in CK. Notably, the levels of delicious and bitter amino acids exhibited significant (*p* < 0.005) increases of 20.187% and 51.987%, respectively. The delicious amino acids content in the bamboo shoots treated with T2 showed a significant increase of 9.020% compared to CK (*p* < 0.005). The contents of flavoring amino acids in the bamboo shoots treated with T3 did not differ significantly from those in the CK group (**Figure 4a**).

Further analysis of the proportions of each flavoring amino acid revealed that, relative to the CK treatment, the proportion of delicious amino acids in the bamboo shoots across all three treatments decreased, with a significant (*p* < 0.005) reduction of 5.389% observed in the T1 treatment. Conversely, the proportion of bitter amino acids in the bamboo shoots treated with T1 and T3 increased significantly (*p* < 0.005) by 19.598% and 16.398%, respectively (**Figure 4b**). These findings suggest that the application of *Bacillus subtilis*, bamboo charcoal, and their combination significantly influences the contents and proportions of flavoring amino acids in Lei bamboo shoots to varying extents.

#### 3.3.4 Comprehensive evaluation of the quality of Lei bamboo (*Phyllostachys praecox*) shoots under different treatments

**Figure 5:**
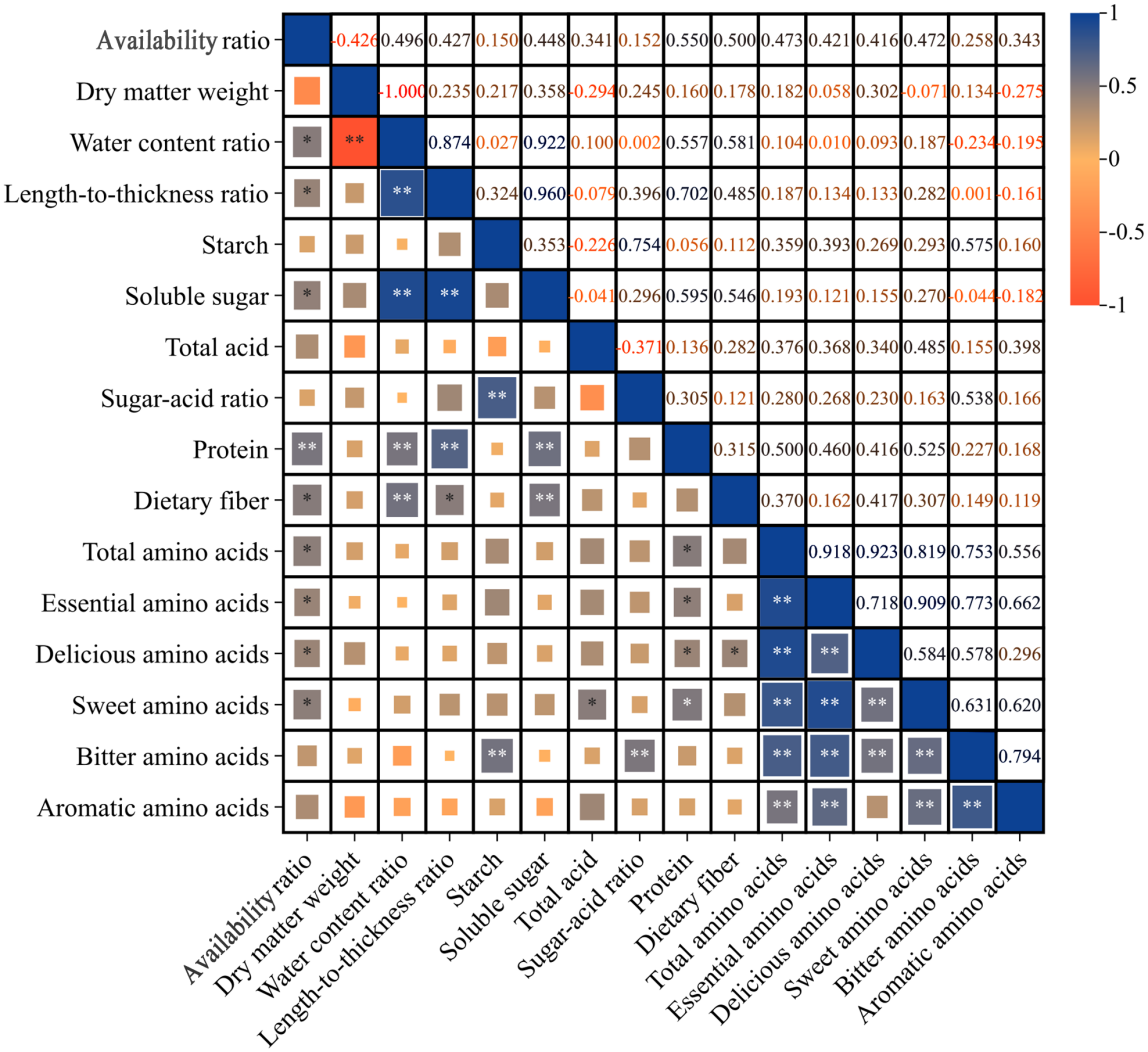
Correlation analysis of various indicators of Lei bamboo (*Phyllostachys praecox*) shoots in different treatments. Asterisks denote statistically significant differences between the groups, as measured by Mann–Whitney rank-sum test (**p < 0.01 and *p < 0.05).

The results of the correlation analysis for each index, as illustrated in **Figure 5**, indicate that the availability ratio was significantly positively correlated with several factors, including water content ratio (0.496), length-to-thickness ratio (0.427), soluble sugar (0.448), dietary fiber (0.500), total amino acids (0.473), essential amino acids (0.421), delicious amino acids (0.416), and sweet amino acids (0.472). Notably, it exhibited an extremely significant positive correlation with protein (0.550). Conversely, the dry matter weight demonstrated an extremely significant negative correlation with the water content ratio (-1.000). Furthermore, the water content ratio was extremely significantly positively correlated with the length-to-thickness ratio (0.874), soluble sugar (0.922), protein (0.557), and dietary fiber (0.581). The length-to-thickness ratio showed a significant positive correlation with dietary fiber (0.485) and an extremely significant positive correlation with both soluble sugar (0.960) and protein (0.702). Starch was extremely significantly positively correlated with the length-to-thickness ratio (0.754) and bitter amino acids (0.575). Additionally, soluble sugar was extremely significantly positively correlated with protein (0.595) and dietary fiber (0.546). The total acid exhibited a significant positive correlation with sweet amino acids (0.485), while the sugar-acid ratio was extremely significantly positively correlated with bitter amino acids (0.538). Protein was significantly positively correlated with total amino acids (0.500), essential amino acids (0.460), delicious amino acids (0.416), and sweet amino acids (0.525). Dietary fiber was significantly positively correlated with delicious amino acids (0.417). The total amino acids were extremely significantly positively correlated with essential amino acids (0.918), delicious amino acids (0.923), sweet amino acids (0.819), bitter amino acids (0.753), and aromatic amino acids (0.556). Essential amino acids were extremely significantly positively correlated with delicious amino acids (0.718), sweet amino acids (0.909), bitter amino acids (0.773), and aromatic amino acids (0.662). Delicious amino acids were extremely significantly positively correlated with sweet amino acids (0.584) and bitter amino acids (0.578). Sweet amino acids were extremely significantly positively correlated with bitter amino acids (0.631) and aromatic amino acids (0.620), while bitter amino acids were extremely significantly positively correlated with aromatic amino acids (0.794). In summary, the data suggests that the various quality indicators of Lei bamboo shoots exhibit overlapping information, making it challenging to evaluate these indicators in isolation. Therefore, it is imperative to process and simplify the indicators that demonstrate higher correlations to improve the reliability of the quality assessment of Lei bamboo.

A principal component analysis was performed on 16 indicators pertaining to the quality of Lei bamboo shoots. It was determined that principal components with eigenvalues exceeding 1 and a cumulative percentage of variance greater than 85% were deemed representative to a certain degree (Bian et al., 2024). In this study, a total of five principal components were identified, with eigenvalues of 4.803, 4.261, 2.295, 1.409, and 1.042, respectively. The corresponding percentages of variance for these components were 30.016%, 26.63%, 14.345%, 8.805%, and 6.509%, leading to a cumulative percentage of variance of 86.305% (**Table 8**). This analysis indicates that the majority of the information encapsulated within these principal components effectively reflects the quality indicators of Lei bamboo shoots.

**Table 8:** Correlation coefficient matrix eigenvalues and cumulative variance contribution.

Composition	Initial Eigenvalue			Square summation of loads extracted		
	Total	Percentage of variance	Accumulates%	Total	Percentage of variance	Accumulates%
1	4.803	30.016	30.016	4.803	30.016	30.016
2	4.261	26.63	56.646	4.261	26.63	56.646
3	2.295	14.345	70.991	2.295	14.345	70.991
4	1.409	8.805	79.795	1.409	8.805	79.795
5	1.042	6.509	86.305	1.042	6.509	86.305
6	0.821	5.13	91.435			
7	0.516	3.224	94.659			
8	0.497	3.106	97.765			
9	0.167	1.044	98.809			
10	0.083	0.52	99.329			
11	0.064	0.4	99.729			
12	0.022	0.135	99.863			
13	0.014	0.085	99.948			
14	0.008	0.049	99.998			
15	0	0.002	100			
16	5.88E-16	3.68E-15	100			

The principal component factor loading matrix (**Table 9**) reveals that principal component 1 was predominantly influenced by indicators such as soluble sugar, bitter amino acids, starch, sugar-acid ratio, length-to-thickness ratio, total acid, and total amino acids. Notably, while total acid exhibited a negative loading, the remaining indicators demonstrated positive loadings. Consequently, principal component 1 primarily encapsulated the attributes of sweetness and amino acid accumulation in the bamboo shoots of Lei bamboo. In contrast, principal component 2 was chiefly influenced by amino acid indicators and total acid indicators, both of which exhibit positive loadings, alongside sugar-related indicators that displayed negative loadings. Thus, principal component 2 predominantly reflected the profile associated with amino acid enrichment in the bamboo shoots. Principal component 3 was primarily indicative of the texture and structural characteristics of Lei bamboo shoots, with the bamboo shoots exhibiting a crisper and more tender texture due to a high water content ratio and low dietary fiber content. Principal component 4 was significantly influenced by dietary fiber content (0.620) under positive loading and protein content (-0.732) under negative loading, suggesting a mutual exclusivity among nutritional components. Finally, principal component 5 primarily reflected the availability ratio of bamboo shoots, where a higher availability ratio correlated with increased protein content and decreased acidity.

**Table 9:** Load matrix corresponding to principal components.

Indicators	Principal component				
	1	2	3	4	5
Soluble Sugar	0.776	-0.455	0.168	0.085	-0.210
Bitter Amino Acid	0.766	0.445	0.123	0.271	-0.023
Starch	0.717	-0.327	0.204	0.422	-0.234
Sugar-acid Ratio	0.703	-0.565	0.260	0.096	0.210
Length-to-thickness Ratio	0.697	-0.473	0.302	-0.295	0.002
Total Acid	-0.632	0.565	-0.160	-0.173	-0.415
Total Amino Acids	0.572	0.745	-0.234	0.034	0.049
Essential Amino Acid	0.598	0.745	0.037	-0.073	-0.167
Sweet Amino Acid	0.441	0.743	0.114	-0.108	-0.280
Aromatic Amino Acid	0.177	0.688	0.331	0.183	-0.069
Delicious Amino Acid	0.396	0.549	-0.498	0.050	0.226
Dry Matter Weight	0.471	-0.276	-0.775	-0.132	-0.018
Water Content Ratios	-0.471	0.276	0.775	0.132	0.018
Protein	0.429	0.253	0.090	-0.732	0.371
Dietary Fiber	-0.126	0.127	-0.480	0.620	0.297
Availability Ratios	-0.086	0.468	0.398	0.124	0.578

The principal component scores were computed in accordance with the methodology established by Bai (Bai et al., 2012). The comprehensive score, denoted as F, is derived by weighting the variance percentages of the selected five principal components relative to the total variance. This score serves as an evaluative measure of the overall quality of Lei bamboo shoots subjected to various treatments. The formula for the comprehensive score evaluation model is expressed as F = F1 × 0.30016 + F2 × 0.2663 + F3 × 0.14345 + F4 × 0.08805 + F5 × 0.06509. The results of the scoring and the corresponding rankings are presented in **Table 10**. A higher comprehensive score indicates superior overall quality of Lei bamboo shoots. The findings indicate that the comprehensive quality of the shoots follows the order: T1 > T2 > T3 > CK.

**Table 10:** Quality evaluation results of Lei bamboo (*Phyllostachys praecox*) shoots in different treatments.

Treatments	Principal component score					Comprehensive score	Ranking
	F1	F2	F3	F4	F5		
CK	-1.9042	0.2712	-0.3356	1.3380	-0.6540	-0.4722	4
T1	2.7931	0.5568	0.3365	0.2782	0.2134	1.0733	1
T2	-1.7042	1.4667	-0.3774	-1.1228	0.6777	-0.2298	2
T3	0.8152	-2.2946	0.3765	-0.4933	-0.2372	-0.3712	3

### 3.4 Correlation analysis between the underground system of Lei bamboo (*Phyllostachys praecox*) and the quality of its shoots

**Figure 6:**
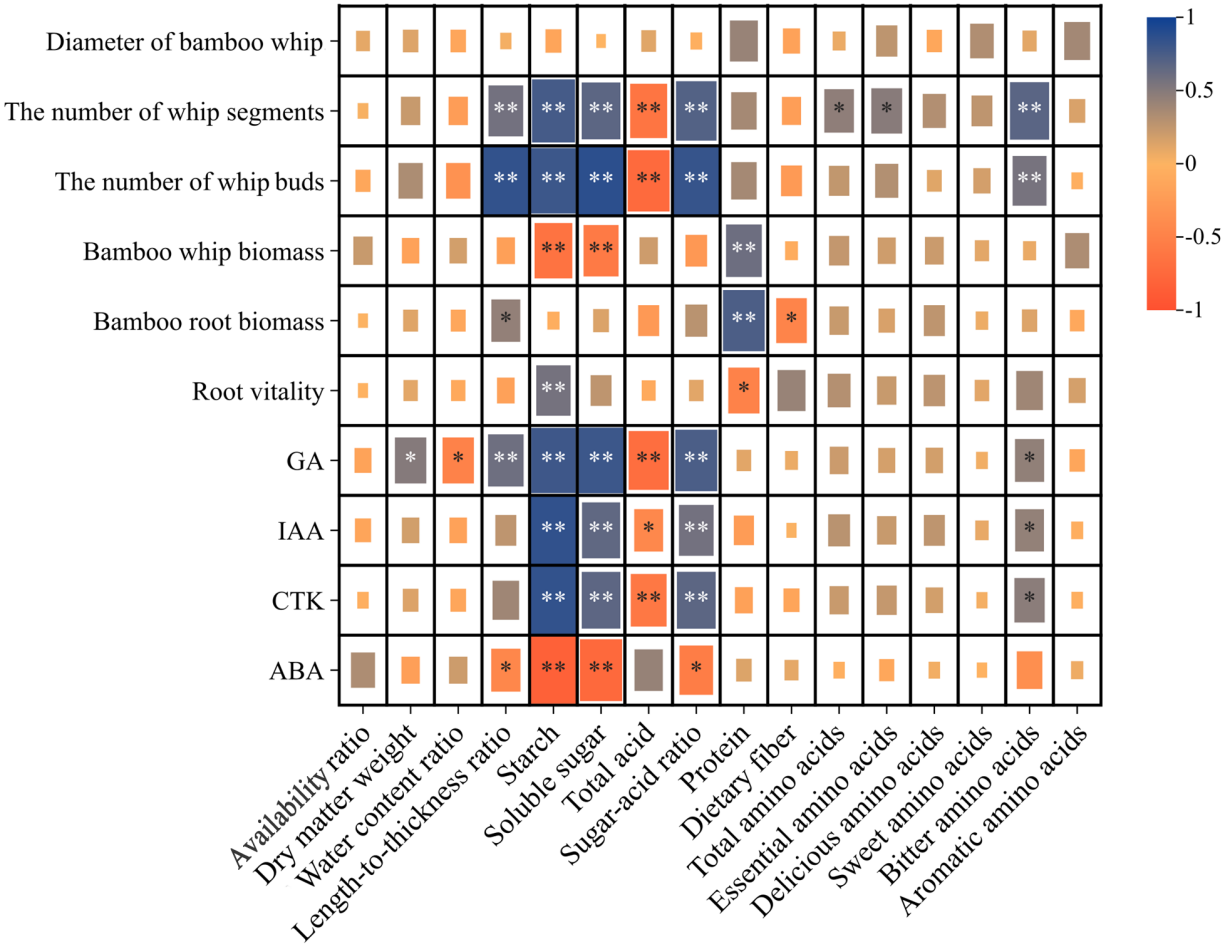
Correlation Analysis of the underground system of Lei bamboo (*Phyllostachys praecox*) and the quality of its shoots. Asterisks denote statistically significant differences between the groups, as measured by Mann–Whitney rank-sum test (**p < 0.01 and *p < 0.05).

The Pearson correlation analysis presented in **Figure 6** indicates that starch, soluble sugar, sugar-acid ratio, and bitter amino acids are positively correlated with the number of whip segments, the number of buds, GA, IAA, and CTK, while showing negative correlations with ABA. In contrast, total acid displays the opposite trend. Additionally, protein is positively correlated with both bamboo whip and root biomass, while negatively correlated with root vitality.

## 4 Discussion

### 4.1 The application of *Bacillus subtilis* and bamboo charcoal influences the endogenous hormone levels in Lei bamboo (*Phyllostachys praecox*)

The findings show that *Bacillus subtilis* (T1) significantly enhanced soil fertility, notably by raising total organic carbon by 54.2% and nitrogen by 43.7%. These improvements create a nutrient-rich rhizosphere that supports hormone production and root function, reflected in increased levels of growth-promoting hormone. Central to this intricate physiological regulation driven by microorganisms is the enhancement of specific enzymatic reactions and metabolic pathways. Similar patterns have been observed in other microbial systems. For instance, research on *Pseudomonas putida* demonstrates that this strain effectively breaks down organic sulfur by optimizing its enzymatic machinery (Ai et al., 2025; Qiao et al., 2024), highlighting the precise adaptability of microbial metabolic pathways to particular environmental substrates. Drawing a parallel to this, it is probable that *Bacillus subtilis* synthesizes hormone precursors directly by modulating its enzyme systems through comparable mechanisms, such as the involvement of IAA N-acetyltransferase in IAA production (Shao et al., 2015; Putrie et al., 2021) or terpene synthase related to GA synthesis (Pramastya et al., 2020). Additionally, its metabolic processes may enhance the expression of key hormone biosynthesis enzymes in plant roots, including GA20-oxidase (Hu et al., 2024) and YUCCA family proteins (Samaras et al., 2022). This complex network of enzymatic reactions initiated by microbial activity represents a potential biochemical mechanism regulating hormones within the subterranean system, ultimately influencing the quality of bamboo shoots.

Comparable mechanisms have been observed in wheat and tomato, where *Bacillus subtilis* lowered stress-related ABA accumulation while maintaining growth-promoting hormones (Hu et al., 2024; Tahir et al., 2017; Lastochkina et al., 2023). The ability of beneficial microbes to improve plant stress tolerance has also been confirmed at the molecular level in other crops. For instance, Wang, Y et al. (2023) discovered that the heat shock protein TaHSP17.4 in wheat plays a crucial role in enhancing the plant's overall stress resistance by interacting with the chaperone protein TaHOP. This chaperone helps stabilize protein structures during stress, preventing misfolding and denaturation. In this study, the hormone balance remodeling induced by *Bacillus subtilis* (specifically reducing ABA and maintaining growth-promoting hormone levels) is likely to synergize with the activation of similar host plant molecular protection mechanisms. Microbial partners may regulate plant responses not only through hormone signaling but also by boosting the plant's physiological resilience at the molecular level, for example, by increasing the expression of important functional proteins like heat shock proteins, thereby creating a more comprehensive strategy for stress adaptation.

The application of bamboo charcoal (T2) has been found to reduce the levels of GA, IAA, and CTK in Lei bamboo rhizomes, while simultaneously raising ABA levels. Adding a large amount of bamboo charcoal significantly changed the soil's chemical properties around the bamboo. It notably increased soil pH by 3.6%.Although the overall rise in soil pH was minimal, for the roots of Lei bamboo, which have long adapted to a highly acidic environment (pH 3.618 in CK treatment), any movement into the neutral pH could constitute a significant disturbance of the rhizosphere environment, triggering a stress response, triggering a stress response. Additionally, bamboo charcoal, known for its strong adsorption capacity, can capture various organic and inorganic substances (Lou et al., 2019). Its large specific surface area can substantially change the physicochemical conditions of the rhizosphere by absorbing root exudates or beneficial microbial metabolites. This disruption of the plant's niche may be perceived as an abiotic stress. Furthermore, applying bamboo charcoal increases the expression of genes related to the biosynthesis and signaling of ABA and other defense-related hormones like jasmonic acid and auxin (He et al., 2024). Under different stress conditions, Lei bamboo tends to decrease the production of growth-promoting hormones (IAA, GA, CTK) and increase the synthesis of stress-related hormones (ABA) (Ghosh et al., 2018; Takahashi et al., 2022). Moreover, bamboo charcoal raised soil total organic carbon (TOC) by 16.2%, had a smaller effect on nitrogen levels (+9.2%), and only moderately enhanced soil nutrients. This aligns with the observed trade-offs between aboveground biomass growth and nutrient allocation, reflecting a survival strategy where resources are shifted from growth toward defense and adaptation.

Unlike the carbon and nitrogen indices, neither treatment had a significant impact on the soil total phosphorus (TP) content (**Table 3**). This could be due to several reasons: First, phosphorus tends to become fixed in the soil, especially in the acidic soil studied here, where it readily binds with iron and aluminum ions to form compounds that are poorly soluble, reducing its effectiveness. As a result, it is difficult to significantly increase the total phosphorus pool through biomodifiers in a short period. Second, the duration of this experiment may not have been long enough to capture notable changes in the phosphorus pool. Additionally, although the application of *Bacillus subtilis* and bamboo charcoal improved the soil environment, their effect on phosphorus activation is likely more evident in the increase of available phosphorus rather than an immediate change in total phosphorus content, a finding also reported in similar studies on Lei bamboo forest soil (Ni et al., 2024). Therefore, the relative stability of total phosphorus does not exclude the possibility that the treatments positively influence phosphorus availability and its uptake and utilization by roots.

### 4.2 The application of *Bacillus subtilis* and bamboo charcoal influences the morphological and biomass characteristics of the underground system of Lei bamboo (*Phyllostachys praecox*)

*Bacillus subtilis* plays a pivotal role in nutrient activation, promotion of root growth, and induction of plant immunity. During its metabolic processes, *Bacillus subtilis* is capable of synthesizing plant hormone analogues, which directly stimulate cell division and differentiation within the meristematic tissue of bamboo whips. This stimulation facilitates the formation of whip segments and the germination of whip buds (Wang et al., 2024). Furthermore, *Bacillus subtilis* secretes organic acids and enzymes that convert insoluble phosphorus and potassium in the soil into forms that are readily absorbable by plants (Jensen et al., 2024). As a nutrient storage organ, the bamboo whip exhibits significant biomass accumulation in nutrient-rich environments. *Bacillus subtilis* also accelerates the decomposition of organic matter, such as bamboo leaves and rice husks, leading to the production of humic acid substances (Seemakram et al., 2023). These substances not only provide a carbon source for bamboo whip growth but also integrate trace elements, thereby enhancing the bioavailability of essential micronutrients such as iron and zinc. This process operates on principles similar to other methods that use microbes to stabilize carbon. For instance, Xing et al. (2025) demonstrated that adding external substances can greatly improve the humification process during the thermophilic co-composting of food waste residues, while also reducing greenhouse gas emissions. Their results indicate that by controlling microbial activity, unstable organic materials can be transformed into stable humus, enabling carbon storage and lessening environmental impact. Similarly, in this study, the use of *Bacillus subtilis* likely acts as a "biological additive," not only speeding up mulch decomposition but also potentially directing its transformation into stable humus. This may explain the notable increase in soil total organic carbon observed. Thus, applying *Bacillus subtilis* serves not just as an agricultural practice but also as a sustainable management approach that combines soil carbon sequestration with emission reduction, significantly enhancing its environmental sustainability role in bamboo forest ecosystems. Additionally, bamboo charcoal offers physical structural support and acts as a carrier for slow-release nutrients. Its abundant nano-pores and large specific surface area contribute to increased soil porosity and improved water retention capacity (Huynh et al., 2021). In a moist and loose environment, the resistance of bamboo whip expansion decreases, resulting in accelerated biomass accumulation. Moreover, the bamboo charcoal is rich in basic ions, such as calcium and magnesium, which can help regulate soil pH, mitigate acidification, and enhance the cell division activity of bamboo roots. The application of *Bacillus subtilis* and bamboo charcoal not only fulfills the fertility requirements of Lei bamboo but also establishes a low-stress, high-vitality underground habitat, thereby laying the groundwork for the enhancement of bamboo shoot quality.

The bamboo whip biomass exhibits a negative correlation with GA, IAA and CTK, while demonstrating a positive correlation with ABA. This phenomenon can be attributed to the fact that during the rapid extension phase of bamboo whips, the limited availability of nutrients and energy is preferentially directed towards cell division and longitudinal growth within the flagellar meristem, leading to an increase in the number of whip segments. The elevation of IAA levels facilitates cell elongation but concurrently inhibits cell lignification and the thickening of secondary walls. GA primarily promote internode elongation, serving as a critical regulatory factor for the elongation of bamboo shoot stems, whereas CTK encourage the differentiation of lateral buds into whip buds. Collectively, these hormones enhance the number of whip segments and buds. However, this process demands substantial resources, thereby compromising the radial growth of bamboo whips. The phenomenon can be interpreted as an adaptive strategy for resource optimization. This strategy entails a hormone-mediated rapid spatial expansion that prioritizes short-term biomass reduction in favor of enhancing long-term population expansion potential.

### 4.3 The application of *Bacillus subtilis* and bamboo charcoal enhances the quality of Lei bamboo (*Phyllostachys praecox*) shoots

The application of *Bacillus subtilis* has been shown to enhance the levels of GA, IAA and CTK in Lei bamboo, while also increasing the contents of starch, soluble sugars, and bitter amino acids in bamboo shoots. The synergistic effects of these growth hormones significantly facilitate the processes of cell division and elongation in bamboo shoots, which also explained the phenomenon that the length-to-thickness ratio of Lei bamboo shoots was the largest under *Bacillus subtilis* treatment. This developmental process necessitates substantial energy and carbon skeletons for the synthesis of new cellular components. In response to hormonal signals, the degradation of starch is expedited by hydrolytic enzymes, leading to the production of soluble sugars such as glucose, which contributes to the elevated contents of soluble sugars (Li et al., 2022). Soluble sugars serve not only as energy sources but also function as osmotic regulators and signaling molecules, thereby assisting in the maintenance of cell turgor pressure and supporting rapid cellular elongation. Sugar and hormones jointly regulate the growth and development of bamboo shoots (Zheng et al., 2025). The rapidly growing Lei bamboo shoots are typically characterized by their tenderness and juiciness. The increased levels of IAA, GA and CTK, while promoting growth, may also trigger or activate secondary metabolic pathways as a form of pre-adaptation or defense mechanism against potential stressors, such as herbivory and pest infestations. This activation leads to an accumulation of precursor substances, specifically bitter amino acids (Gamuyao et al., 2017). Enhanced metabolic activity results in increased carbon and nitrogen fluxes, which correlates with a rise in the contents of soluble sugars and bitter amino acids at certain developmental stages. Starch serves as the primary carbon reserve in bamboo shoots and may increase to satisfy the substantial energy and material demands associated with rapid growth. Although starch levels may decrease due to degradation, they may also temporarily rise in hormone-driven metabolic sites, such as bamboo shoots, due to the swift influx of photosynthetic products.

The application of bamboo charcoal has been shown to enhance the levels of ABA and total acid content in Lei bamboo. The substantial specific surface area and porous structure of bamboo charcoal create an optimal environment for soil microorganisms. Additionally, bamboo charcoal has the capacity to adsorb and gradually release organic matter and nutrients within the soil, thereby providing a sustained source of energy and nutrients for microbial populations. As microorganisms decompose and utilize these organic materials and nutrients, they metabolize and produce significant quantities of organic acids, including acetic acid, lactic acid, and succinic acid (Deng et al., 2023). A portion of these organic acids is absorbed by the root system of Lei bamboo and accumulates in the bamboo shoots, which directly contributes to the observed increase in total acid content. Furthermore, ABA functions as an “alarm hormone” that enables plants to respond to adverse environmental conditions such as drought, elevated temperatures, and salinity. An increase in ABA levels prompts the closure of stomata, thereby reducing water loss while simultaneously decreasing CO_2_ intake, which can inhibit photosynthesis (Khaleghnezhad et al., 2021). This reduction in photosynthetic activity leads to a decrease in sugar synthesis and induces a shift in cellular metabolism toward a “catabolic” state, enhancing glycolysis and producing pyruvate (Lara-Núñez et al., 2024). The signaling pathways associated with ABA also promote the synthesis of protective compounds, which typically contribute to the overall acid content in plants (Mousavi and Shabani, 2019).

The application of *Bacillus subtilis* and bamboo charcoal individually has been shown to enhance total amino acids content in the Lei bamboo (Phyllostachys praecox) shoots. Conversely, their combined application results in a reduction of total amino acids content, which is basically consistent with the lack of synergistic effect found by Andrea et al. in the combination of *Bacillus siamensis* and biochar (Andrea et al., 2025). *Bacillus subtilis* enhanced soil total organic carbon (TOC) and total nitrogen (TN), boosted bamboo root vitality, and encouraged root growth, which together improved the nitrogen absorption efficiency of Lei bamboo. This aligns with the observed rise in amino acid and protein levels in the aboveground sections of Lei bamboo, demonstrating that microbial-induced activation of soil nutrients translated into better bamboo shoot quality. Additionally, substances secreted by *Bacillu*s *subtilis* can trigger metabolic pathways inside bamboo shoot cells, leading to increased amino acid accumulation (Gao et al., 2023).Bamboo charcoal, characterized by its porous structure, adsorbs and gradually releases essential nutrients such as nitrogen and phosphorus, thereby minimizing nutrient loss and enhancing fertilizer utilization efficiency. Its alkaline nature can also ameliorate acidic soil conditions, fostering microbial activity, which indirectly supports the availability of substrates necessary for amino acid synthesis. Interestingly, the combined application of *Bacillus subtilis* and bamboo charcoal significantly decreased the amino acid content in bamboo shoots by 8.841%. This effect might be explained by the alteration of the soil microbial community in Lei bamboo caused by these additions (Wang et al., 2021; Yan et al., 2024). This conclusion is supported by research on other systems. Ren et al. (2025) demonstrated that *Bacillus subtilis* can enhance nutrient uptake and water use efficiency by optimizing the structure of the rhizosphere microbial community. Their study shows how proactive microbial remodeling can directly lead to improved resource utilization. In this study, the addition of bamboo charcoal may significantly alter the physicochemical characteristics of Lei bamboo rhizosphere microenvironment, potentially resulting in a microbial community structure that hinders efficient nutrient transfer to bamboo shoots. In other words, the effectiveness of *Bacillus subtilis* is highly influenced by the microenvironment it inhabits. When combined with bamboo charcoal, the original ecological niche is disrupted, possibly shifting the microbial community optimization focus from “enhancing plant nutrition” to “microbial community restructuring”. This shift could explain why the beneficial effects seen with *Bacillus subtilis* alone were negated when used together with bamboo charcoal, ultimately leading to a reduction in amino acid accumulation. Moreover, the large surface area and strong adsorption properties of bamboo charcoal might bind *Bacillus subtilis* or its secreted substances, such as antibiotics, enzymes, and growth hormones, thereby lowering their availability. Another possibility is that the increased biomass observed with bamboo charcoal raised the nutrient requirements of Lei bamboo, causing nitrogen to be redirected from amino acid accumulation in the bamboo shoots toward supporting individual plant growth.

### 4.4 The specific processes and mechanisms of *Bacillus subtilis* and bamboo charcoal affecting the quality of Lei bamboo (*Phyllostachys praecox*) shoots: “soil chemistry - hormone - morphology and biomass - quality” cascade mechanism

**Figure 7:**
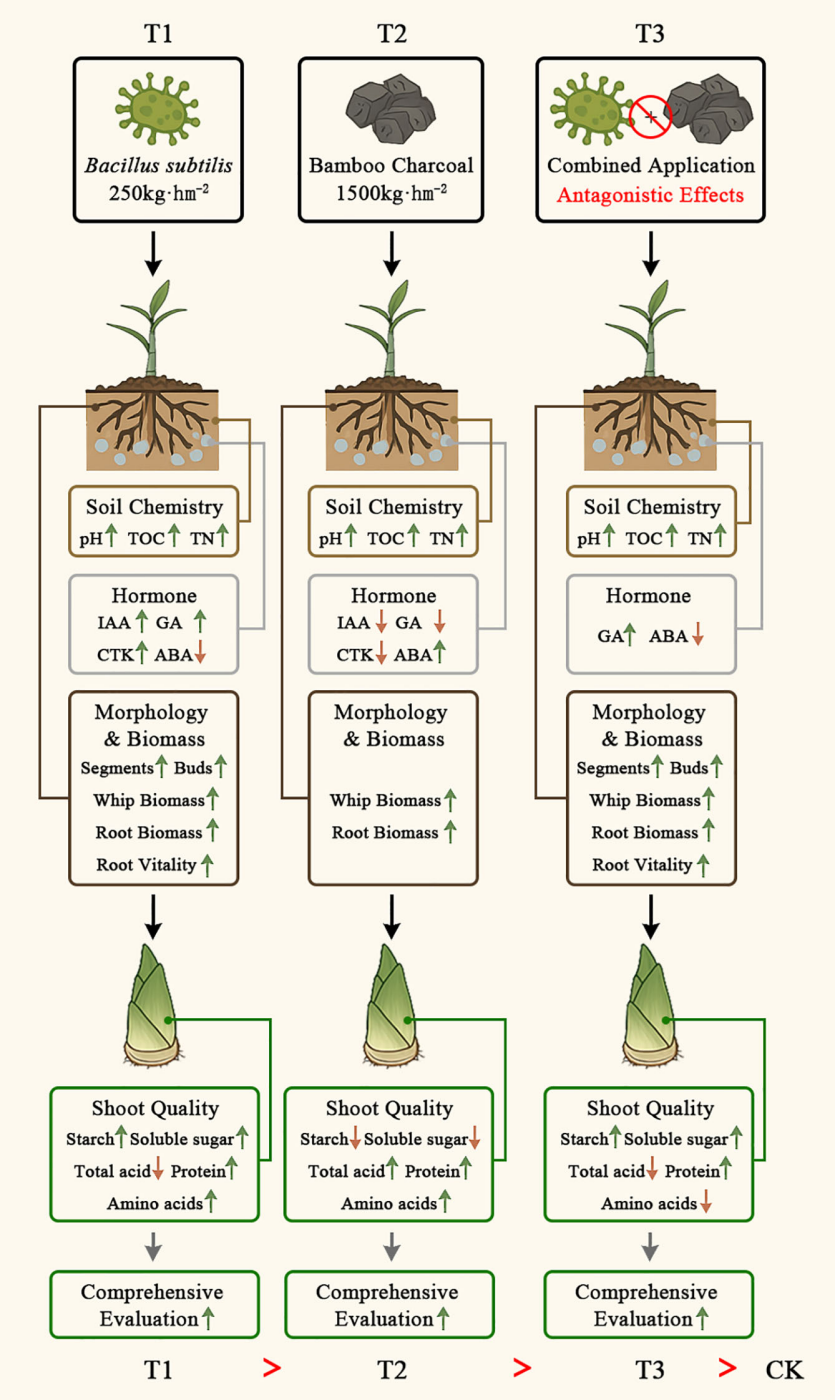
Mechanism of *Bacillus subtilis* and Bamboo Charcoal on Lei Bamboo (*Phyllostachys praecox*) Shoot Quality: A Soil Chemistry-Hormone-Morphology and Biomass-Quality Cascade.

This research, combining examinations of soil chemistry, Lei bamboo (*Phyllostachys praecox*) physiology, and the quality of Lei bamboo shoots, uncovered the specific processes and mechanisms through which *Bacillus subtilis* and bamboo charcoal influence Lei bamboo shoot quality. These results reveal a new “soil chemistry - hormone - morphology and biomass - quality” cascade mechanism (**Figure 7**). This pathway is initiated by *Bacillus subtilis* functioning as a “soil regulator”, enhancing soil TOC and TN levels to establish a nutrient-rich, low-stress rhizosphere environment. Conversely, bamboo charcoal serves as a “physical amendment” and a mild “stress inducer”, altering soil pH and physical structure. These soil modifications subsequently provoke hormonal reprogramming in the underground system of Lei bamboo: *Bacillus subtilis* fosters a growth-promoting hormonal balance characterized by elevated levels of GA, IAA, and CTK, alongside reduced ABA, whereas bamboo charcoal induces stress-related hormonal responses marked by increased ABA and decreased GA, IAA, and CTK. The altered hormone regulates the development of the whip and root system in Lei bamboo, leading to either an increase in the number of segments and buds (T1 and T3) or enhanced whip and root biomass (T2). Ultimately, these morphological and physiological changes in the underground system precondition the accumulation of key quality constituents in bamboo shoots, including sugars and starches (T1), as well as total acids and amino acids (T2). Furthermore, the model provides an explanation for the antagonistic effects observed under combined application (T3), indicating that the effectiveness of *Bacillus subtilis* largely depends on the rhizosphere environment of Lei bamboo. Merely combining it with bamboo charcoal actually have negative effects.

This study has certain limitations. First, the field experiments were carried out at a single test site in the Yuhang area of Hangzhou, so the reported optimal effects of *Bacillus subtilis* and bamboo charcoal on improving Lei bamboo shoot quality still require further validation in other climatic zones (such as drier or colder regions) or different soil types (like neutral or alkaline soils). Second, the experiment used bamboo charcoal with a particle size of 40–80 mesh based on common product specifications, without systematically examining how bamboo charcoal of varying particle sizes (finer or coarser) might differ in effect. Future research should involve multi-location trials across diverse ecological regions and thoroughly investigate how key physical properties of bamboo charcoal (such as particle size and porosity) relate to its agricultural functions, aiming to develop a more broadly applicable and refined fertilization strategy. Future research should also combine soil microbiome sequencing, enzyme activity assessments, and isotopic tracing of nitrogen and carbon flows to confirm the interconnectedness between soil chemistry, hormone regulation, and aboveground metabolism. Additionally, it should examine how nitrogen uptake efficiency and microbial competition influence bamboo shoot quality. Simultaneously, modifying the surface characteristics of bamboo charcoal to decrease its adsorption of microbial metabolites could offer a way to enhance its interaction with *Bacillus subtilis* in bamboo forest ecosystems, while also assessing the economic viability of implementing these methods in large-scale bamboo cultivation.

## 5 Conclusion

In summary, *Bacillus subtilis* and bamboo charcoal serve as effective soil conditioners that enhance the quality of Lei bamboo (*Phyllostachys praecox*) shoots, each operating through distinct mechanisms. The principal theoretical contribution of this study is the development of a new “soil chemistry - hormone - morphology and biomass - quality” cascade mechanism. The model offers a mechanistic insight of how the underground system regulates the quality of aboveground bamboo shoots, with hormones serving as the central regulatory node.

Practically, the study offers clear guidance for precise fertilization of Lei bamboo shoots: 1) To produce high-end fresh bamboo shoots with optimal taste — characterized by a high sugar-to-acid ratio, sweetness, and a crisp, juicy texture — applying 250 kg·hm^-2^ of *Bacillus subtilis* alone is recommended. This approach enhances sugar accumulation through hormonal regulation, yielding high-quality shoots; 2) To develop a unique flavor profile, such as increased amino acid content for richer taste, 1500 kg·hm^-2^ of bamboo charcoal can be applied, but this will raise acidity and reduce sweetness; 3) Avoid simply mixing *Bacillus subtilis* with bamboo charcoal, as this combination may cause antagonistic effects that lower amino acid contents and overall quality, wasting resources.

Future studies should aim to confirm whether this model applies across different ecological regions and explore ways to mitigate antagonism, such as modifying bamboo charcoal, achieving synergistic benefits and support sustainable, intensive bamboo forest management.

## Data Availability

The raw data supporting the conclusions of this article will be made available by the authors, without undue reservation.

## References

[B1] Ai C. Zhang X. Xue S. Sun P. (2025). Optimization and mechanistic investigation of organic sulfur removal from bituminous coal by *Pseudomonas putida*. J. Environ. Chem. Eng. 13, 118437. doi: 10.1016/j.jece.2025.118437

[B2] Amin M. Ahmed M. (2023). Biological control of onion white rot disease using potential *Bacillus subtilis* isolates. Egypt. J. Biol. Pest Control 33, 1–7. doi: 10.1186/s41938-023-00673-4

[B3] Andrea C. Jorge C. Fernando G. (2025). Boosting olive plant defense systems against *Verticillium dahliae*: integrating biochar and *Bacillus siamensis* for sustainable disease management. Ind. Crops Prod. 233, 121395. doi: 10.1016/j.indcrop.2025.121395

[B4] Ankush D. Deepti D. Manish K. Phani K. Lal S. (2023). Bamboos as a cultivated medicinal grass for industries: a systematic review. Ind. Crops Prod. 203, 117210. doi: 10.1016/j.indcrop.2023.117210

[B5] Bai S. Bi J. Wang P. Gong L. Y. Wang X. (2012). Research on comprehensive evaluation of apple quality based on principal component analysis. Food Sci. Technol. 37, 54–57. doi: 10.13684/j.cnki.spkj.2012.01.034

[B6] Belincanta C. Botelho G. Ornellas T. Zappelini J. Guerra M. (2021). Characterization of the endophytic bacteria from *in vitro* cultures of *Dendrocalamus asper* and *Bambusa oldhamii* and assessment of their potential effects in *in vitro* co-cultivated plants of *Guadua chacoensis* (*Bambusoideae, Poaceae*). In Vitro Cell. Dev. Biol. Plant 58, 122–132. doi: 10.1007/s11627-021-10204-1

[B7] Bian L. Liang D. Fan M. Wu H. Zhou B. Yao W. . (2024). Analysis and comprehensive evaluation of nutrient components in bamboo shoots of different clones of *Chimonobambusa utilis*. J. Nanjing For. Univ. Nat. Sci. Ed. 48, 159–167. doi: 10.12302/j.issn.1000-2006.202206046

[B8] Bigatton E. Castillejo M. Ayoub I. Baldessari J. Bruno M. Archilla M. . (2024). Plant growth promoting rhizobacteria (PGPR): impact on peanut flowering, seed physical quality, and yield determination (*Arachis hypogaea L.*). Ind. Crops Prod. 219, 119024. doi: 10.1016/j.indcrop.2024.119024

[B9] Blake C. Kovács Á. Christensen M. (2021). Molecular aspects of plant growth promotion and protection by. Bacillus subtilis. Mol. Plant Microbe Interact. 34, 15–25. doi: 10.1094/MPMI-08-20-0225-CR, PMID: 32986513

[B10] Chen J. Hu J. Wang Y. Seah R. Zhang S. Zhu Y. . (2023). Bamboo charcoal affects soil properties and bacterial community in tea plantations. Open Life Sci. 18, 20220681. doi: 10.1515/biol-2022-0681, PMID: 37589012 PMC10426720

[B11] Deng S. Mbukwa D. Gui R. (2023). Effects of aeration treatments on root and rhizome growth parameters of *Phyllostachys violascens* (Lei bamboo) under intensive cultivation: a field study. Sci. Total Environ. 900, 165738. doi: 10.1016/j.scitotenv.2023.165738, PMID: 37495156

[B12] Deng Y. Han R. Ding N. Zhou S. (2023). Metabolic engineering and fermentation optimization strategies for producing organic acids of the tricarboxylic acid cycle by microbial cell factories. Bioresour. Technol. 379, 128986. doi: 10.1016/j.biortech.2023.128986, PMID: 37001700

[B13] Fallah N. Pang Z. Zhang C. Tayyab M. Yang Z. Lin Z. . (2023). Complementary effects of biochar, secondary metabolites, and bacteria biocontrol agents rejuvenate ratoon sugarcane traits and stimulate soil fertility. Ind. Crops Prod. 202, 117081. doi: 10.1016/j.indcrop.2023.117081

[B14] Gamuyao R. Ayano M. Minami A. Kojima M. Higashiyama T. Nagai K. . (2017). Hormone distribution and transcriptome profiles in bamboo shoots provide insights on bamboo stem emergence and growth. Plant Cell Physiol. 58, 702–716. doi: 10.1093/pcp/pcx023, PMID: 28204696

[B15] Gao J. Bai Y. Zheng H. Cai M. Cheng Z. Mu C. . (2023). Integrative analysis of exogenous auxin mediated plant height regulation in Moso bamboo (*Phyllostachys edulis*). Ind. Crops Prod. 200, 116852. doi: 10.1016/j.indcrop.2023.116852

[B16] Gayathri M. Sharanya R. Renukadevi P. Varagur G. Amalendu G. Saranya N. (2025). Genomic configuration of *Bacillus subtilis* (NMB01) unveils its antiviral activity against *Orthotospovirus arachinecrosis* infecting tomato. Front. Plant Sci. 16. doi: 10.3389/fpls.2025.1517157, PMID: 40104030 PMC11913681

[B17] Ghosh D. Gupta A. Mohapatra S. (2018). Dynamics of endogenous hormone regulation in plants by phytohormone secreting rhizobacteria under water-stress. Symbiosis 77, 265–278. doi: 10.1007/s13199-018-00589-w

[B18] Guleria S. Kumar M. Khan A. Kaushik R. (2021). Plant hormones: physiological role and health effects. J. Microbiol. Biotechnol. Food Sci. 11, e1147. doi: 10.15414/JMBFS.1147

[B19] Haleema T. Sowmyalakshmi S. Anja G. Donald L. (2025). *Bacillus* and *Paenibacillus* as plant growth-promoting bacteria in soybean and cannabis. Front. Plant Sci. 16. doi: 10.3389/fpls.2025.1529859, PMID: 40525084 PMC12169014

[B20] He T. Chen L. Wu Y. Wang J. Wu Q. Sun J. . (2024). Combined metabolome and transcriptome analyses of maize leaves reveal global effect of biochar on mechanisms involved in anti-herbivory to *Spodoptera frugiperda*. Metabolites 14, 498. doi: 10.3390/metabo14090498, PMID: 39330505 PMC11433984

[B21] Hu J. Guo Z. Chen S. Fan L. He Q. (2023). Shoot nutrition and flavoring variation in two *Phyllostachys* species: does the quality of availability bamboo shoot diaphragm and flesh differ? Foods 12, 1180. doi: 10.3390/foods12061180, PMID: 36981107 PMC10048675

[B22] Hu Q. Xiao Y. Liu Z. Huang X. Dong B. Wang Q. (2024). *Bacillus subtilis* QM3, a plant growth-promoting rhizobacteria, can promote wheat seed germination by gibberellin pathway. J. Plant Growth Regul. 43, 2682–2695. doi: 10.1007/s00344-024-11298-8

[B23] Huynh B. Nguyen T. Tran V. (2021). The change of sandy soil properties after adding charcoal produced from a traditional kiln in the Mekong Delta, Viet Nam. Sci. J. Tra Vinh Univ. 1, 109–115. doi: 10.35382/18594816.1.42.2021.698

[B24] Jensen C. Pang J. Gottardi M. Kračun S. Svendsen B. Nielsen K. . (2024). *Bacillus subtilis* promotes plant phosphorus (P) acquisition through P solubilization and stimulation of root and root hair growth. Physiol. Plant 176, e14338. doi: 10.1111/ppl.14338, PMID: 38740528

[B25] Jiang Z. Lin S. Hu X. Wang Q. Ning S. Song Z. (2024). Application of magnetized ionized water and *Bacillus subtilis* improved saline soil quality and cotton productivity. Plants 13, 2458. doi: 10.3390/plants13172458, PMID: 39273942 PMC11397375

[B26] Khaleghnezhad V. Yousefi A. Tavakoli A. Farajmand B. Mastinu A. (2021). Concentrations-dependent effect of exogenous abscisic acid on photosynthesis, growth and phenolic content of *Dracocephalum moldavica* L. under drought stress. Planta 253, 127. doi: 10.1007/s00425-021-03648-7, PMID: 34036415 PMC8149364

[B27] Lara-Núñez A. Guerreo-Molina E. Vargas-Cortez T. Vázquez-Ramos J. (2024). Interplay of CDKs and cyclins with glycolytic regulatory enzymes PFK and PK. J. Plant Physiol. 303, 154378. doi: 10.1016/j.jplph.2024.154378, PMID: 39541719

[B28] Lastochkina O. Yuldashev R. Avalbaev A. Allagulova C. Veselova S. (2023). The contribution of hormonal changes to the protective effect of endophytic bacterium *Bacillus subtilis* on two wheat genotypes with contrasting drought sensitivities under osmotic stress. Microorganisms 11, 2955. doi: 10.3390/microorganisms11122955, PMID: 38138099 PMC10745732

[B29] Li W. Huang D. Wang B. Hou X. Zhang R. Yan M. . (2022). Changes of starch and sucrose content and related gene expression during the growth and development of Lanzhou lily bulb. PloS One 17, e0262506. doi: 10.1371/journal.pone.0262506, PMID: 35015792 PMC8752016

[B30] Li Q. Huang Z. Zhong Z. Bian F. Zhang X. (2024). Integrated genomics and transcriptomics provide insights into salt stress response in *Bacillus subtilis* ACP81 from Moso bamboo shoot (*Phyllostachys praecox*) processing waste. Microorganisms 12, 285. doi: 10.3390/microorganisms12020285, PMID: 38399690 PMC10893186

[B31] Lou L. Huang Q. Lou Y. Lu J. Hu B. Lin Q. (2019). Adsorption and degradation in the removal of nonylphenol from water by cells immobilized on biochar. Chemosphere 228, 676–684. doi: 10.1016/j.chemosphere.2019.04.151, PMID: 31063914 PMC6771920

[B32] Lu Y. Yuan X. Lin X. Wei F. (2012). Endogenous hormone changes during floral bud morphological differentiation of *Phyllostachys violascens*. J. Zhejiang A F Univ. 29, 161–165. doi: 10.11833/j.issn.2095-0756.2012.02.002

[B33] Lv X. Liu S. Cao Y. Wu H. Zhang C. Huang B. . (2025). Multiwalled carbon nanotubes promoted biofilm formation and rhizosphere colonization of *Bacillus subtilis* Tpb55. J. Agric. Food Chem. 73, 7087–7098. doi: 10.1021/acs.jafc.4c10818, PMID: 39992185

[B34] Mehmood T. Azeem M. Anwaar N. Gul S. Aftab A. Javed S. . (2023). Application of Bacillus subtilis for the alleviation of salinity stress in different cultivars of wheat (*Triticum aestivum* L.). Agronomy 13, 437. doi: 10.3390/agronomy13020437

[B35] Mousavi S. Shabani L. (2019). Rosmarinic acid accumulation in *Melissa officinalis* shoot cultures is mediated by ABA. Biol. Plant 63, 418–424. doi: 10.32615/BP.2019.057

[B36] Ni H. Zhao J. Yang Z. (2024). Effects of compound fertilizer decrement and water-soluble humic acid fertilizer application on soil properties, bacterial community structure, and shoot yield in Lei bamboo (*Phyllostachys praecox*) plantations in subtropical China. Forests 15, 400. doi: 10.3390/f15030400

[B37] Pramastya H. Xue D. Abdallah I. Setroikromo R. Quax W. (2020). High level production of amorphadiene using *Bacillus subtilis* as an optimized terpenoid cell factory. New Biotechnol. 60, 159–167. doi: 10.1016/j.nbt.2020.10.007, PMID: 33148534

[B38] Putrie R. Aryantha I. Iriawati Antonius S. (2021). The structure characteristic of IAA n-acetyl-transferase enzyme produced by two species of bacteria (*Bacillus subtilis* and *Bacillus amyloliquefaciens*). IOP Conf. Ser. Earth Environ. Sci. 762, 12054. doi: 10.1088/1755-1315/762/1/012054

[B39] Qian Y. Jia J. Chen Z. Wang K. Li P. Gao P. . (2025). Environmental drivers and transcriptomic variations shaping Lei bamboo shoots across cultivation regions. Front. Plant Sci. 16. doi: 10.3389/fpls.2025.1565665, PMID: 40235921 PMC11997477

[B40] Qiao P. Ning L. Chen J. Tang Y. Zhao R. Chen G. . (2024). The critical roles of propanethiol oxidoreductase and sulfide-quinone oxidoreductase in the propanethiol catabolism pathway in. Pseudomonas putida, S–1 90, e0195923. doi: 10.1128/aem.01959-23, PMID: 38193681 PMC10880595

[B41] Qin D. Sun Y. Chen Y. Guo X. Gong Q. Miao S. . (2023). Biocontrol endophytes *Bacillus subtilis* R31 influence the quality, transcriptome and metabolome of sweet corn. PeerJ 11, e14967. doi: 10.7717/peerj.14967, PMID: 36883062 PMC9985898

[B42] Ramakrishna W. Mahapatra S. Yadav R. (2022). *Bacillus subtilis* impact on plant growth, soil health and environment: Dr. Jekyll and Mr. Hyde. J. Appl. Microbiol. 132, 3543–3562. doi: 10.1111/jam.15480, PMID: 35137494

[B43] Ren X. Chen L. Zhu Q. Hou Y. Zhang J. Jin A. . (2023). Bamboo charcoal mediated plant secondary metabolites biosynthesis in tomato against South American tomato pinworm (*Tuta absoluta*). Front. Sustain. Food Syst. 7. doi: 10.3389/fsufs.2023.1101151

[B44] Ren P. Zhou B. Bi Y. Chen X. Yao S. Yang X. (2025). *Bacillus subtilis* can promote cotton phenotype, yield, nutrient uptake and water use efficiency under drought stress by optimizing rhizosphere microbial community in arid area. Ind. Crops Prod. 227, 120784. doi: 10.1016/j.indcrop.2025.120784

[B45] Saini H. Arya I. (2016). Bioremediation of oil polluted soil: effect on hill bamboo (*Drepanostachyum falcatum*) plant emergence and height. J. Agric. Biotechnol. Sustain. Dev. 8, 46–52. doi: 10.5897/JABSD2016.0269

[B46] Samaras A. Kamou N. Tzelepis G. Karamanoli K. Menkissoglu-Spiroudi U. Karaoglanidis G. (2022). Root transcriptional and metabolic dynamics induced by the plant growth promoting rhizobacterium (PGPR) *Bacillus subtilis* Mbi600 on cucumber plants. Plants 11, , 1218. doi: 10.3390/plants11091218, PMID: 35567219 PMC9102019

[B47] Sarti G. Galelli M. Cristóbal-Miguez J. Cárdenas-Aguiar E. Chudil H. García A. . (2024). Inoculation with biofilm of *Bacillus subtilis* is a safe and sustainable alternative to promote tomato (*Solanum lycopersicum*) growth. Environments 11, 54. doi: 10.3390/environments11030054

[B48] Seemakram W. Suebrasri T. Khaekhum S. Ekprasert J. Boonlue S. (2023). Enhancement of integrated sugarcane trash managements by co-inoculation of cellulolytic microorganisms for sustaining soil fertility. Sugar Tech 25, 925–937. doi: 10.1007/s12355-023-01250-7

[B49] Shao J. Li S. Zhang N. Cui X. Zhou X. Zhang G. . (2015). Analysis and cloning of the synthetic pathway of the phytohormone indole-3-acetic acid in the plant-beneficial *Bacillus amyloliquefaciens* SQR9. Microb. Cell Fact. 14, 130. doi: 10.1186/s12934-015-0323-4, PMID: 26337367 PMC4558970

[B50] Siska I. Natsir A. Ako A. Utamy R. (2024). Bamboo activated charcoal on phytochemical substances and quality of cassava leaves (*Manihot utilissima*). Bio Web Conf. 123, 1005. doi: 10.1051/bioconf/202412301005

[B51] Tahir H. Gu Q. Wu H. Raza W. Hanif A. Wu L. . (2017). Plant growth promotion by volatile organic compounds produced by *Bacillus subtilis* SYST2. Front. Microbiol. 8. doi: 10.3389/fmicb.2017.00171, PMID: 28223976 PMC5293759

[B52] Takahashi Y. Munemasa S. Waadt R. Seller C. Schroeder J. Hsu P. (2022). Plant hormone regulation of abiotic stress responses. Nat. Rev. Mol. Cell Biol. 23, 680–694. doi: 10.1038/s41580-022-00479-6, PMID: 35513717 PMC9592120

[B53] Thapa U. Ansari Z. Ramesh S. Anbalagan K. Rabi A. (2024). Plant hormones and growth regulators: mechanisms, interactions, and agricultural applications. Agric. Arch. 3, 11–20. doi: 10.51470/agri.2024.3.3.11

[B54] Umemura M. Takenaka C. (2014). Retranslocation and localization of nutrient elements in various organs of moso bamboo (*Phyllostachys pubescens*). Sci. Total Environ. 493, 845–853. doi: 10.1016/j.scitotenv.2014.06.078, PMID: 25000580

[B55] Wang L. Bao C. Liang N. Cheng Q. Mao W. Yang H. (2024). All-year high IAA and ABA contents in rhizome buds may contribute to natural four-season shooting in woody bamboo *Cephalostachyum pingbianense*. Plants 13, 410. doi: 10.3390/plants13030410, PMID: 38337943 PMC10857254

[B56] Wang Y. Li W. Du B. Li H. (2021). Effect of biochar applied with plant growth-promoting rhizobacteria (PGPR) on soil microbial community composition and nitrogen utilization in tomato. Pedosphere 31, 872–881. doi: 10.1016/S1002-0160(21)60030-9

[B57] Wang W. Li B. Liu D. Han L. Zhao L. Zhang Y. . (2023). *Bacillus subtilis* promotes cucumber growth and quality under higher nutrient solution by altering the rhizospheric microbial community. Plants 12, 298. doi: 10.3390/plants12020298, PMID: 36679013 PMC9862796

[B58] Wang Y. Yu T. Wang C. Wei J. Zhang S. Liu Y. . (2023). Heat shock protein TaHSP17.4, a TaHOP interactor in wheat, improves plant stress tolerance. Int. J. Biol. Macromol. 246, 125694. doi: 10.1016/j.ijbiomac.2023.125694, PMID: 37414309

[B59] Wu J. Wu Q. Ruan Y. Cai G. Li S. Tu J. (2022). The effect of bamboo charcoal application on soil nutrients and heavy metals in rice. Phyton 91, 1245–1256. doi: 10.32604/phyton.2022.019599

[B60] Xing X. Wang R. Guo Y. Li X. Zhu Z. Ouyang C. . (2025). Effects of exogenous additives on thermophilic co-composting of food waste digestate: coupled response of enhanced humification and suppressed gaseous emissions. Energy Environ. Sustain. 1, 100046. doi: 10.1016/j.eesus.2025.100046

[B61] Yan W. Liu Y. Malacrinò A. Zhang J. Cheng X. Rensing C. . (2024). Combination of biochar and PGPBs amendment suppresses soil-borne pathogens by modifying plant-associated microbiome. Appl. Soil Ecol. 193, 105162. doi: 10.1016/j.apsoil.2023.105162

[B62] Yin X. Li Z. Yang S. Luo Y. (2023). Mixing bamboo charcoal with vinasse fertilizer to remediate the phytotoxicity of quinclorac to tobacco. Arch. Agron. Soil Sci. 70, 1–14. doi: 10.1080/03650340.2023.2291445

[B63] Zhao S. Kang J. Wang J. (2025). Elucidating the biodegradation mechanisms of atrazine and nicosulfuron by *Bacillus subtilis* KC01: insights into strain functionality and soil application potential. Ind. Crops Prod. 234, 121601. doi: 10.1016/j.indcrop.2025.121601

[B64] Zhao H. Li J. Li X. Hu Q. Guo X. Wang Y. . (2025). Response of soil organic carbon and bacterial community to amendments in saline-alkali soils of the Yellow River Delta. Eur. J. Soil Sci. 76, e70147. doi: 10.1111/ejss.70147

[B65] Zheng H. Bai Y. Xu J. Xie Y. Cheng Z. Gao J. (2025). Transcriptome and phosphoproteomics provides potential insights into how sucrose regulates the growth of bamboo shoots. Ind. Crops Prod. 224, 120439. doi: 10.1016/j.indcrop.2024.120439

[B66] Zhou J. Huo X. Cheng K. Cai Z. Meng P. Wang T. . (2022). Bioorganic fertilizer promotes pakchoi growth and shapes the soil microbial structure. Front. Plant Sci. 13. doi: 10.3389/fpls.2022.1040437, PMID: 36426155 PMC9679507

[B67] Zou L. Yan H. Peng L. Zhao G. Xia M. Chen H. . (2024). The impacts of plant hormones on the growth and quality of sprouts. Food Bioprocess Technol. 17, 2913–2942. doi: 10.1007/s11947-023-03216-9

